# Molecular evolution of dentin phosphoprotein among toothed and toothless animals

**DOI:** 10.1186/1471-2148-9-299

**Published:** 2009-12-23

**Authors:** Dianalee A McKnight, Larry W Fisher

**Affiliations:** 1 Craniofacial and Skeletal Diseases Branch, NIDCR, NIH, DHHS, Bethesda MD 20892 USA

## Abstract

**Background:**

Dentin sialophosphoprotein (DSPP) is the largest member of the SIBLING family and is the most abundant noncollagenous protein in dentin. DSPP is also expressed in non-mineralized tissues including metabolically active ductal epithelia and some cancers. Its function, however, is poorly defined. The carboxy-terminal fragment, dentin phosphoprotein (DPP) is encoded predominantly by a large repetitive domain that requires separate cloning/sequencing reactions and is, therefore, often incomplete in genomic databases. Comparison of DPP sequences from at least one member of each major branch in the mammalian evolutionary tree (including some "toothless" mammals) as well as one reptile and bird may help delineate its possible functions in both dentin and ductal epithelia.

**Results:**

The BMP1-cleavage and translation-termination domains were sufficiently conserved to permit amplification/cloning/sequencing of most species' DPP. While the integrin-binding domain, RGD, was present in about half of species, only vestigial remnants of this tripeptide were identified in the others. The number of tandem repeats of the nominal SerSerAsp phosphorylation motif in toothed mammals (including baleen whale and platypus which lack teeth as adults), ranged from ~75 (elephant) to >230 (human). These repeats were not perfect, however, and patterns of intervening sequences highlight the rapidity of changes among even closely related species. Two toothless anteater species have evolved different sets of nonsense mutations shortly after their BMP1 motifs suggesting that while cleavage may be important for DSPP processing in other tissues, the DPP domain itself may be required only in dentin. The lizard *DSPP *had an intact BMP1 site, a remnant RGD motif, as well as a distinctly different Ser/Asp-rich domain compared to mammals.

**Conclusions:**

The DPP domain of *DSPP *was found to change dramatically within mammals and was lost in two truly toothless animals. The defining aspect of DPP, the long repeating phosphorylation domain, apparently undergoes frequent slip replication and recombination events that rapidly change specific patterns but not its overall biochemical character in toothed animals. Species may have to co-evolve protein processing mechanisms, however, to handle increased lengths of DSP repeats. While the RGD domain is lost in many species, some evolutionary pressure to maintain integrin binding can be observed.

## Background

Dentin sialophosphoprotein (*DSPP*) is the most centromeric of five tandem SIBLING (Small Integrin-Binding LIgand N-linked Glycoprotein) genes that are clustered together within 375 kb at 4q22.1 in humans [1]. Due to shared intron/exon structures and exon-specific motifs, all of the SIBLINGs have been proposed to have been sequentially derived from a single ancient gene by duplication [1]. To our knowledge, there are no reports of any of the five SIBLING genes being located outside of their tandem gene cluster in any species. The SIBLING family has recently been suggested to be included as a distinct subclass (called the "acidic" gene cluster) of a larger cluster of genes, the secretory calcium-binding phosphoprotein (SCPP) [2]. The inclusion of the SIBLINGs gene family into the SCPP gene cluster is due to the predicted ability of all members to bind calcium ions and the observation that they all tend to remain clustered together on a single chromosome for many species studied to date. All SIBLINGs are expressed by cells responsible for the assembly and/or maintenance of mineralized type I collagen matrices (bone and dentin) and for many years most were thought to be limited to such calcified tissues. The human *DSPP *gene is comprised of five exons, the last four of which encode the ~1300 amino acid protein. Two other SIBLINGs, bone sialoprotein (BSP) and osteopontin (OPN or SPP1), have been shown by nuclear magnetic resonance (NMR) analysis to be completely flexible in solution [1] and DSPP is expected to also be unstructured in solution. Like many other flexible proteins in biology, members of the SIBLING family are thought to function by binding to a number of different proteins using short motifs that remain conserved while many of the intervening amino acids are permitted to change with time. A conserved MQXDD peptide motif encoded within the largest and most 3' exon of *DSPP *is where bone morphogenic protein-1 (BMP1) is hypothesized to cleave the protein (at the amino-terminus of the first aspartic acid) into the amino-terminal dentin sialoprotein (DSP) and the carboxy-terminal dentin phosphoprotein (DPP) in at least type I collagen matrix-producing cells [3-6]. BMP1 has been shown to cleave another SIBLING member, dentin matrix protein-1 (DMP1), at this same motif [6,7]. (This protease also releases the C-propeptide from type I collagen and processes several other secreted bioactive proteins such as biglycan and members of the TGFβ superfamily [6]). Human DPP contains the classic integrin-binding tripeptide, arginine-glycine-aspartate (RGD, a hallmark of the SIBLING gene family) found 26 amino acids carboxy-terminal to the BMP1-cleavage site.

Most (~85%) of the human DPP sequence is comprised of a phosphorylated serine/aspartate-rich repeat domain. The size of this repeat domain is unique to DSPP and appears to have developed through many separate expansions of a nominal 9-basepair microsatellite-like repeat encoding the phosphorylation tripeptide motif, serine-serine-aspartate (SSD). It was established long ago that tandem repeats of short DNA sequences are unstable. They are susceptible to slip replication errors as well as unequal recombination events during meiosis such that the total number of the repeats can change in relatively few generations. Humans, having recently (in evolutionary terms) gone through a population bottleneck, are a genetically restricted species but we have recently shown that the 3' 1 kb portion of DPP's repeat domain in 188 chromosomes selected from geographically diverse humans had 37 different haplotypes due to various combinations of 37 SNPs and 20 insertions/deletions (indels) [8]. According to the HapMap project http://hapmap.ncbi.nlm.nih.gov/, the human *DSPP *gene appears to currently be within a recombination cold spot suggesting that these recent indels were likely caused by slip replication errors. Similar repeat-length differences have also been described for pig DPP [5]. Because most humans and other non-inbred mammals have many different DPP haplotypes involving indels, most individual animals are heterozygotic with respect to this gene and analysis of their DPP domains can usually be accomplished only by cloning and sequencing of each allele. Furthermore, the combination of repeat length polymorphisms and the inherent difficulties of sequencing long stretches of microsatellite-like repeats have resulted in sequence gaps (N's) in the DPP domain of many animal genome sequencing projects. Indeed, deciphering of the DPP domain within future genome projects may remain problematical as the next generation technologies of high-throughput genome analysis are relying on the compilation of short (40-100 bp) sequencing reactions that cannot be uniquely assembled for long stretches of microsatellite-like repeats.

The function of DSPP has not been fully defined. DSPP was originally thought to be expressed solely in dentin where it is by far the most abundant noncollagenous protein entrapped within the mineralized matrix. Due to its high degree of phosphorylation that results in a high calcium ion-binding capacity, DPP has long been hypothesized to directly nucleate and/or control the growth of hydroxyapatite crystals within the type I collagen dentin matrix. Independent studies estimate that ~50% of the serines in DPP are phosphoserines [5,9,10] making this perhaps the most acidic and hydrophilic protein in mammals. In humans, all verified cases of non-syndromic dentinogenesis imperfecta and dentin dysplasia have been shown to be due to dominant negative mutations in the *DSPP *gene, many resulting from -1 frameshifts within DPP's repeat domain [8,11,12]. In mice, the *Dspp*-null mutation is recessive and homozygotic null mice have a dentinogenesis imperfecta phenotype with incomplete mineralization of the dentin matrix [13]. Recent work showing that the *Dspp*-null mouse was not completely rescued by expression of the DSP domain alone [14] suggests that the DPP domain plays an important direct or indirect role in dentin matrix production and/or mineralization.

All of the SIBLINGs including DSPP, however, have also been shown to be expressed in the epithelial cells of ducts such as salivary gland, kidney, and sweat glands of both primates and rodents [15-18]. Because they are not expressed within the passive ducts of the lachrymal gland, we have hypothesized that expression of DSPP and all other SIBLINGs may be important in metabolically active ducts [15] and their associated cancers [19], perhaps by interacting with specific members of the matrix metalloproteinase family [20]. Interestingly, mutations in *DSPP *that are known to cause dominant negative defects in human dentin have not been reported to affect these soft tissues. The homozygotic *Dspp*-null mouse, however, has been reported to exhibit aberrant organogenesis in the kidney and the lung although no adult phenotype was reported to be associated with these soft tissue [21].

In this report, the sequences of the DPP in lizard, chicken, and 26 mammalian species (including at least one member of each major branch of the mammalian phylogenetic tree) were analyzed to investigate through an evolutionary lens, the retained motifs and thereby the possible functions of this unusual protein. We examined toothless animals to address the hypothesis that the DPP portion of DSPP may perform an irreplaceable function only in dentin.

## Methods

### Identification of DPP in mammals, chicken and lizard

As specifically denoted in Table 1, the DPP sequences used in this study were obtained by one of the following methods: 1) previously annotated and complete sequences found in noted databases or identified as being related to *DSPP *on the UCSC genome browser http://genome.ucsc.edu; 2) derived from scaffolds on the UCSC browser as open reading frames and appropriately located within the SIBLING-SPARCL1 gene cluster but not specifically identified as *DSPP*/DPP; 3) manual scanning of all open reading frames in conjunction with the SplicePort program http://spliceport.cs.umd.edu/ for conserved motifs within possible exons 5' to *DMP1 *and up to the *SPARCL1 *gene when present; 4) PCR amplification of genomic DNA of species whose *DSPP*/DPP domain was partially complete and available on one of the databases but required cloning and sequencing to complete DPP's repetitive domain; and 5) PCR amplification/cloning/sequencing of genomic DNA from species with no published DPP sequences. Novel sequences were submitted to GenBank (FJ204896-FJ204920 and FJ204922-FJ204927).

**Table 1:** Species utilized in this study and the sources of DNA or sequence data

Species	Source	Sample Identifier	GenBank ID/Comments
*Tamandua mexicana* (Anteater, Tamandua)	Leona Chemnick, Beckman Center for Conservations Research/CRES	KB12462	GenBank:FJ204923
*Myrmecophaga tridactyla* (Anteater, Giant)	Leona Chemnick, Beckman Center for Conservations Research/CRES	OR831	GenBank:FJ204926
*Dasypus novemcinctus* (Armadillo)	American Type Culture Collection	CRL-6009, Trachea cells	GenBank:FJ204896
*Myotis austroriparius* (Bat)	David A. Ray, Dept of Biology, West Virginia University,	Maus M8133 or M8135	No sequence obtained
*Felis catus* (Cat)	Novagen	69235	GenBank:FJ204897 GenBank:FJ204898
*Pan troglodytes* (Chimpanzee)	Coriell Cell Repositories, Coriell Institute for Medical Research	NA03448	GenBank:FJ20489 GenBank:FJ204900
*Bos taurus* (Cow)	Novagen	69231	GenBank:FJ204901 GenBank:FJ204902
*Odocoileus virginianus* (Deer)	Highlands Land Association, Fort Hill, PA	7X57-139 gr	GenBank:FJ204903
*Canis lupus familiaris* (Dog)	Novagen	69234	GenBank:FJ204904
*Tursiops truncatus* (Dolphin, Bottlenose)	Southwest Fisheries Science Center, La Jolla, CA	Z 57948, MBB1	GenBank:FJ204927
*Delphinus delphis* (Dolphin, Short-Beaked Common)	Southwest Fisheries Science Center, La Jolla, CA	Z 23148	GenBank:FJ204905 GenBank:FJ204906
*Tachyglossus aceleatus* (Echidna)	Frank Grützner, School of Molecular & Biomed. Sci., Univ. Adelaide, Australia		No sequence obtained
*Loxodonta africana* (Elephant, African)	Stergios-Orestis Kolokotronis, American Museum of Natural History, NY, NY	AR11-105098	GenBank:FJ204909 GenBank:FJ204910
*Elephas maximus* (Elephant, Asian)	Stergios-Orestis Kolokotronis, American Museum of Natural History, NY, NY	AR9-105095	GenBank:FJ204907 GenBank:FJ204908
*Gorilla gorilla* (Gorilla)	Coriell Cell Repositories, Coriell Institute for Medical Research	NG05251	GenBank:FJ204911 GenBank:FJ204912
*Cavia porcellus* (Guinea Pig)	Pamela Gehron Robey, NIDCR, NIH, Bethesda, MD	bone marrow stromal cells, P8	GenBank:FJ204913
*Erinacaeus concolor* (Hedgehog, Eastern)	Godfrey M. Hewitt, University of East Anglia, Norwich, UK		No sequence obtained
*Erinacaeus europeus* (Hedgehog, European)	Godfrey M. Hewitt, University of East Anglia, Norwich, UK		No sequence obtained
*Equus caballus* (Horse)	Roger Smith & Jayesh Dudhia, The Royal Veterinary College, London, UK	UCSC Genome Browser, Sep 2007, equCab2	
*Homo sapiens* (Human)	GenBank		NM_014208
*Dipodomys ordii* (Kangaroo Rat)	UCSC Genome Browser	Jul 2008, dipOrd1	
*Lemur catta* (Lemur)	Coriell Cell Repositories, Coriell Institute for Medical Research	NA7099	GenBank:FJ204914
*Mammuthus primigenius* (Mammoth)	Mammoth Genome Project, PSU	http://mammoth.psu.edu	
*Trichechus manatus latirostris* (Manatee)	Robert K. Bonde, U.S. Geological Survey, Florida Integrated Sci. Center	CCR-007-009, 10, 11	GenBank:FJ204915 GenBank:FJ204916
*Callithrix jacchus* (Marmoset)	UCSC Genome Browser	June 2007, calJac1	
*Mus musculus* (Mouse)	GenBank		NC_000071
*Monodelphis domestica* (Opossum, Gray short-tailed)	UCSC Genome Browser	Jan 2006, monDom4	
*Didelphis marsupialis virginiana* (Opossum, Virginia)	American Type Culture Collection	CRL-1840, cortex kidney cells	GenBank:FJ204917
*Manis tricuspis* (Pangolin)	Leona Chemnick, Beckman Center for Conservations Research/CRES	KB16041	No sequence obtained
*Sus scrofa* (Pig)	Novagen	69230	GenBank:FJ204919
*Ornithorhynchus anatinus* (Platypus)	Frank Grützner, School of Molecular & Biomed. Sci., Univ. Adelaide, Australia		GenBank:FJ204918
*Oryctolagus cuniculus* (Rabbit)	Pamela Gehron Robey, NIDCR, NIH, Bethesda, MD	bone marrow stromal cells, P5	GenBank:FJ204920
*Rattus norvegicus* (Rat)	GenBank		NM_012790
*Macaca mulatta* (Rhesus Macaque)	UCSC Genome Browser	Jan 2006, rheMac2	
*Blarina brevicauda* (Shrew, Northern short-tailed)	Mr. Jack Hubley, Lititz, PA		No sequence obtained
*Sorex araneus* (Shrew, Common)	UCSC Genome Browser	June 2006, sorAra1	
*Sorex ornatus* (Shrew, Ornate)	Leona Chemnick, Beckman Center for Conservations Research/CRES	KB13763	No sequence obtained
*Ateles geoffroyi* (Spider Monkey)	Coriell Cell Repositories, Coriell Institute for Medical Research	NG05352	GenBank:FJ204922
*Spermophilus tridecemlineatus* (Squirrel)	UCSC Genome Browser	Feb 2008, speTri1	
*Tarsier syrichta* (Tarsier)	UCSC Genome Browser	Aug 2008, tarSyr1	
*Balaena mysticetus* (Whale, Bowhead (baleen))	Southwest Fisheries Science Center, La Jolla, CA	Z 11215, BMYS981022	GenBank:FJ204924 GenBank:FJ204925
*Balaenoptera physalus* (Whale, Fin)	Southwest Fisheries Science Center, La Jolla, CA	Z 25397, DSJ010903.01	No sequence obtained
*Physeter macrocephalus* (Whale, Sperm)	Southwest Fisheries Science Center, La Jolla, CA	Z 49068, MAC050820.05	No sequence obtained

### PCR, Cloning, and Sequencing of DPP

Genomic DNA was obtained from a variety of generous sources noted in Table 1. Genomic DNA (50-300 ng) was thermocycled with Platinum^® ^Taq DNA-Polymerase (Invitrogen) using 0.1 mM dNTP, 1.5 mM MgCl_2_, and 0.2 μM each of forward (AGTCCATGCAAGGAGATGATCC) and reverse (CTAATCATCACTGGTTGAGTGG) primers. Standard PCR conditions: 94°C for 5 min followed by 35 cycles (94°C for 30 sec, 55°C for 30 sec, 3 min at 72°C) and a final 5 min at 72°C. Opossum, manatee, whale, dolphin, and giant anteater required optimized conditions (Additional File 1). Amplicons were gel-purified, cloned into pCR4-TOPO (Invitrogen), and sequenced by the NIDCR DNA Sequencing Core facility as previously described [8]. All species were sequenced using the M13 forward and reverse primers associated with the cloning vector while some species required the use of optimized DPP-associated primers described in Additional File 2. To separate true allelic differences (SNPs) from DNA replication errors cause by the Taq DNA polymerase itself, the sequence of each allele was verified by the analysis of at least 3 independent clones.

## Results and Discussion

### Identification and sequencing of DPP domain in mammals

**Figure 1:**
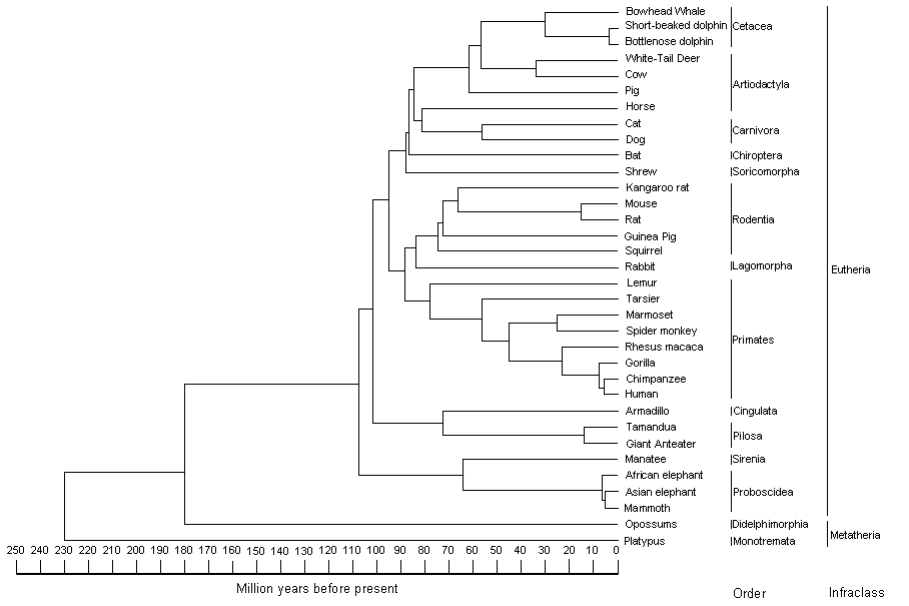
**Phylogeny and divergence timescales of mammalian species whose DPP sequences were compared**. The phylogenetic and divergence time estimates are compilations of results reported in other molecular genetic studies and were based on genes other than *DSPP *[22-32].

At the start of this study, the complete DPP sequences of 4 species (human, chimp, mouse, and rat) were available in GenBank from a combination of cDNA and genomic results. Comparison of these relatively few amino acid sequences showed that although there was a significant difference among the species, fortunately the domains that define the two ends of translated DPP protein (the amino terminal BMP1-cleavage motif and the end-of-translation domain) were sufficiently conserved to permit the design of a small set of oligonucleotide pairs for priming the PCR reactions. Amplification of DPP from European hedgehog, eastern hedgehog, northern shrew, ornate shrew, sperm whale, as well as three animals that are toothless in adulthood (pangolin, fin whale, and echidna) was unsuccessful suggesting that one or both primer-annealing sites may occasionally become sufficiently unlike any of our current primer-pairs to permit amplification in the PCR reactions. Following the protocols developed for the human *DSPP *studies, the DPP amplicons from 22 species were completely analyzed for at least one DPP-encoding allele. Except for highly inbred species (e.g. guinea pig) two different alleles/haplotypes that usually included both indels and SNPs of DPP were observed in each genomic DNA sample although the repeat domain of both alleles was not always fully sequenced for every species. The spectrum of differences observed between haplotypes from a single species were similar to those described in humans [8] and pigs [5]. These differences were mostly characterized by synonymous C→T transitions and small indels. This study differs, however, from our previous approach of haplotyping many different human DPP alleles. In the current study, we compared differences in DPP motifs/domains among many species using a single allele from each. For example, we looked for characteristic motifs that had remained invariant among human haplotypes such as the integrin-binding RGD motif and asked if this motif was conserved among other species. In general, the longest available allele for each species was arbitrarily used for analysis in this study and if a motif was found to be different in a new species (e.g. loss of RGD), that difference was verified in the second allele. (Data of second allele for species are not shown). Figure 1 is a molecular phylogenetic tree illustrating one combined set of estimates (from a variety of published sources [22-32]) of the relationships of all the species for which at least portions of the DPP domain sequences have been obtained. For 26 mammalian species and the lizard (green anole), the complete DPP sequence was obtained. For two species (bat and horse), we were able to obtain amplicons but we were unsuccessful in compiling a complete sequence of either allele. However, the 5' non-repetitive region of cloned horse DPP was deemed accurate and direct sequencing of the bat amplicon yielded sequence information used in some analyses.

### Conservation of the BMP1-cleavage domain in DPP

**Figure 2:**
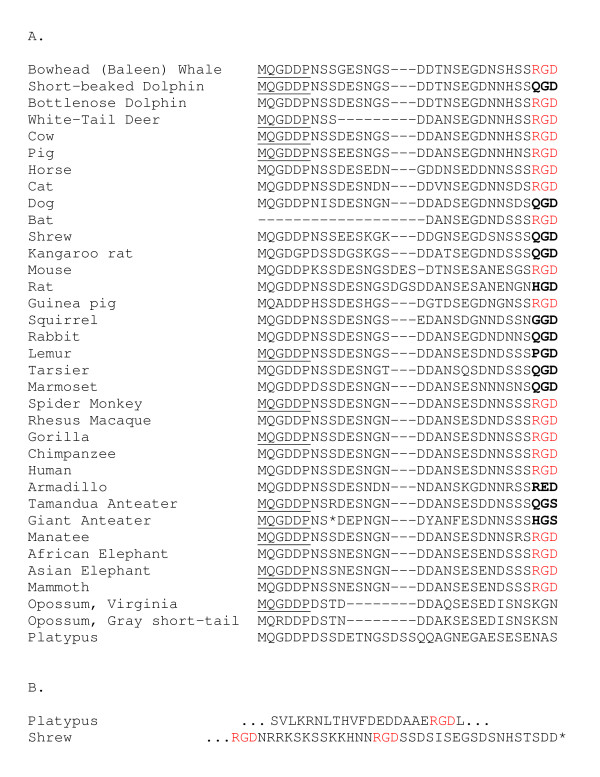
**Alignment of DPP's deduced amino acid sequences from the BMP1-cleavage domain through the RGD/vestigial integrin-binding motifs**. (A) Note the conservation of the BMP1/tolloid-related protease cleavage domain (MQXDD). The underlined MQGDDP sequences were directly encoded by the 5' PCR primer during the production of an amplicon for that species. Due to direct sequencing of the original amplicon, only a portion of the bat sequence was available. The conserved RGD domains (red) are aligned with the vestigial tripeptides (bold). (B) In addition to the loss of their original RGD domains, the platypus was found to contain an RGD domain in a portion of DSP while the common shrew had two RGD motifs within DPP and 5' to the stop codon (*).

The amino-terminal sequence of DPP has been known for many years to start with aspartate-aspartate-proline (DDP) [9]. This sequence corresponds to the motif, MQG**DDP**, in the deduced sequences of DSPP. The protein encoded by *DSPP*'s 3' neighbor SIBLING gene, *DMP1*, has been shown to be cleaved at the same motif by the tolloid-related metalloprotease, BMP1 [6,7]. Although not yet directly proven, it has been hypothesized that BMP1 will also be the protein that cleaves human DSPP into DSP and DPP. In all of the mammals successfully investigated, including the distantly related monotremes, platypus (~230 million years ago, MYA) and marsupials (opossum, ~180 MYA), have conserved the MQXDD motif suggesting that the ability to separate DSP from DPP using the BMP1/tolloid protease family is retained throughout mammalian evolution. 19 of the 21 (90%) of the mammalian species whose BMP1-cleavage motif was independently sequenced (i.e., not the result of the 5' PCR primer) contained the smallest amino acid, glycine (G), in the variable "X" position (Figure 2A). The guinea pig had the next largest amino acid, alanine (A), and the gray short-tailed opossum had the much larger and charged amino acid, arginine (R). While the presence of an arginine at the -1 position of a tolloid-related protease cleavage site is not novel [33], it is important to eventually verify that DPP isolated from the dentin of short-tailed opossum begins with the classic aspartate-aspartate-proline (DDP) amino acid sequence. Interestingly, the kangaroo rat is noted in the UCSC Genome Bioinformatics browser http://genome.ucsc.edu/ for this region of *DSPP *to have substituted a glycine for the second aspartate (D) resulting in a MQGDG motif. The first aspartate is generally thought to be important for cleavage of nearly all of BMP1's protein substrates [33], but the importance of the second aspartate has not been experimentally tested in either DSPP or DMP1.

For the other 13 mammalian species in this study, the sequences encoding the MQGDD motif were directly the result of the 5' primer used in making the PCR amplicon (underlined in Figure 2A). Therefore without verification by an independent sequencing reaction, these 13 species cannot be used to query the identity of the amino acid in the "X" position. The 8 species whose genomic DNA templates did not result in amplicons may have been the result of: 1) synonymous changes that would affect priming but not coding; 2) nonsynonymous changes that affect both priming and coding; or 3) completely missing BMP1-cleavage and/or end-of-translation motifs. The high degree of conservation of the BMP1-cleavage motif at the protein level in all studied *DSPP *sequences, however, does suggest that separation of the two domains (DSP and DPP) is important to their proper physiological functions in at least one critical tissue throughout the class of mammals. By comparison, the BMP1 cleavage domain in several species' DMP1 sequences predominantly had another relatively small amino acid, serine (S), for the "X" in the motif but other species had the glycine seen in *DSPP *as well as the large polar amino acid, asparagine (N) (data not shown).

### Conservation of the RGD integrin-binding domain in DPP

The RGD tripeptide is one of the major defining motifs of the SIBLING gene family and its presence is necessary but not always sufficient for interaction with specific integrins including αvβ3, αvβ5, αvβ6, αvβ1, α5β1, and α8β1 [34]. For example, an intact RGD within both DMP1 and osteopontin (OPN) was necessary for supporting *in vitro *attachment of specific types of cells expressing αvβ3 integrins while another SIBLING, bone sialoprotein (BSP), was found to support such RGD-dependent attachment through either αvβ3 or αvβ5 integrins [7]. Changing the RGD into the chemically similar KAE amino acids destroyed these interactions. Interestingly, OPN's ability to effectively use the α9β 1 integrin remained cryptic until thrombin cleaved this SIBLING at a highly conserved site near the RGD domain [35]. It is not known at this time if the RGD motif of intact human DSPP can interact with any one or a subset of the RGD-dependent integrins and support cell attachment *in vitro*. It will also be interesting to eventually see if the integrin specificity of such interactions (if any) can be modified by cleavage at the BMP1 site.

While more than half of the mammals had the intact integrin-binding motif within 20-28 amino acids downstream of the BMP1 cleavage site, 16 of the 35 (46%) species lacked the RGD. Most of the species that lost their RGD motif within this region had sequences that were presumably inactive remnants of the tripeptide (Figure 2A). At least one species within the orders Pilosa, Cingulata, Carnivora, Cetacea, Primates, Didelphimorphia, Monotremata, Rodentia, and Soricomorpha, had lost this RGD domain. (When two alleles from a single species were available, the conservation or loss of RGD was always verified in the second allele, data not shown). The diversity of the animals missing the RGD domain suggests that the requirement of DPP to bind to a cell surface through this specific site was present in the ancestral *DSPP *gene but was independently lost many times over mammalian evolution. Although several routes to RGD loss can be observed, the most frequent change was the arginine codon, CGA, changing into a glutamine, CAA. This sense strand G-to-A transition likely reflects the deamination of a methylated CpG dinucleotide to TpG on the antisense strand similar to that previously noted for other CpG motifs within the methylated human DPP repeat [8]. (Deamination of the sense strand CpG motif itself would result in a nonsense codon that would presumably be selected against.)

The appearance of RGD missense mutations widely scattered across the mammalian phylogenetic tree would seem to support a hypothesis of harmless random loss of the integrin-binding property with time. However, two other observations need to also be considered; first, the appearance of one new RGD motif in a DSP-encoding portion of platypus *DSPP *and second, two new RGD tripeptides near the carboxy-terminal end of the European shrew's DPP (Figure 2B). These other RGD motifs caution us to acknowledge that at least some animals may retain the requirement for tethering DSPP to at least one cell type through an RGD at some point during their reproductive life spans. In addition, it has not been empirically shown that those DSPP/DPP proteins lacking the RGD are functionally incapable of binding to integrins or other cell surface proteins that do not require this specific tripeptide. Rat DSPP, for example, has changed the original RGD into the presumably inactive HGD but it also has acquired the REDV sequence within its DSP domain. This tetrapeptide is reported to be involved in specific cell attachment of splice variants of fibronectin [36]. In summary, it can be noted that nearly half of the mammalian species do not require an intact RGD but it is possible that the ability to bind DSPP/DPP to a cell surface may remain under evolutionary constraint in some species.

### Conservation of the Repeat Domain in DPP

**Figure 3:**
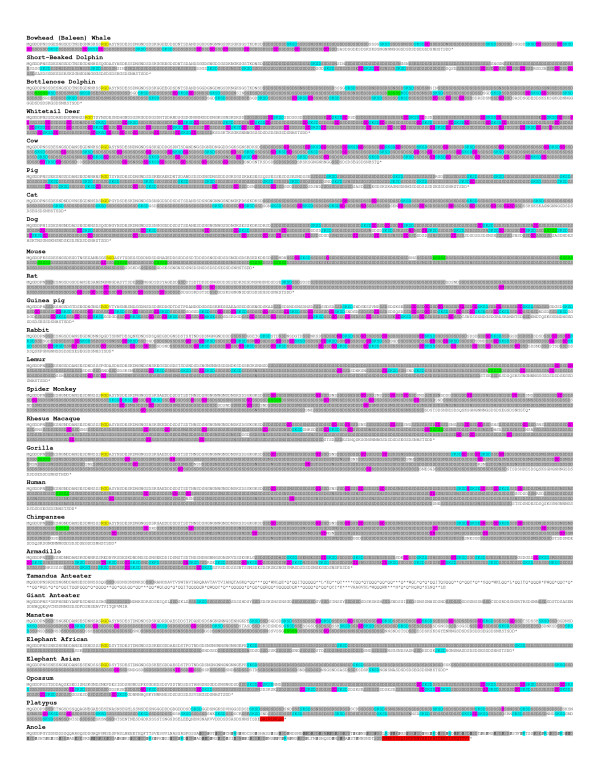
**The DPP sequences of 26 mammalian species and green anole**. Specific motifs are highlighted: SSD-like (highlighted in grey and includes a few simple variations on the tripeptide motif such as SSN and SSE); SKSD-like (blue); RGD (yellow); SSSSS (green); and dipeptides (pink). The extended carboxy-terminal regions for platypus and anole are highlighted in red. Within the anole sequence, serines encoded by TCN-type codons within the repeat domain are in black bold and arginines are highlighted in blue. All serines within the mammalian SSD repeat (except for SKSD) are AGC/T type codons. Stop codons are noted as *. A larger font file of these sequences is available as Additional File 3.

DPP's serine/aspartate-rich domain generally begins ~50-70 amino acids carboxy-terminal to the RGD (intact or remnant) and continues until ~25-35 amino acids before the stop codon. By far the most common element of this domain is the phosphorylation motif, serine-serine-aspartate (SSD). The number of times this nominal tripeptide was repeated differed greatly among mammal species ranging from ~75 for elephant to >230 for humans. (SSD motifs are highlighted in grey in Figure 3 and a larger print version of Figure 3 is also available in Additional File 3.) Except for highly inbred species such as the guinea pig, the exact number of nominal repeats for a single species varied due to allelic differences. For example, among a group of geographically diverse humans, we reported earlier that the length of the repeat domain averaged ~700 amino acids with 95% of haplotypes among 188 chromosomes studied differing by less than 71 amino acids [8]. A DPP repeat length variation was also reported among 8 pigs in which their four haplotypes differed by up to 43 amino acids [5]. Therefore, the sequences in Figure 3, whether derived from database mining or new sequencing, represent only a single allele.

As first discussed in detail by McKnight *et al*. [8], the SSD units in humans are likely to have originated with the primordial 9 bp sequence, AGC AGC GAC, and expanded by a combination of slip replication and unequal recombination events. Single basepair changes, usually attributable to deamination of the methylated CpG and CpApG sequences, explained the most common variations within tripeptides. It was also noted that virtually all of the ~450 serine codons within the human repeat domain were of the AGC/T-type and not TCN-type codons. The only exceptions to the AGC/T-serine codon rule were three SKSD (and the single, related SKSE) tetrapeptides located near the 5' end of the repeat (highlighted in blue in Figure 3). The four SKSD/E tetrapeptides not only used the TCN-type serine codon (TCA), they also accounted for 83% of the positively charged amino acids within the entire ~700 amino acid repeat domain. Because the change of the AGC/T-type serines of a SSD unit would require five sequential single basepair changes to become a KSD, it is reasonable to conclude that this motif was probably introduced a single time into the repeat domain and then duplicated along with portions of the SSD repeat by slip replication and/or recombination events.

As seen by the pattern of the blue-highlighted SKSD tetrapeptides in Figure 3, the total number and general location of these motifs are the same for all higher primates (human, chimpanzee, gorilla, macaque, and spider monkey) although the number of nominal SSD repeats between them differs to some extent. This difference is probably due to slip replication events expanding or contracting the number of the microsatellite-like SSD repeats similar to that seen within the modern human population. Interestingly, the more distantly related primate, lemur, had additional (eight in total) SKSD motifs. This expansion/contraction of a segment of SKSD-containing sequences has apparently occurred by either slip replication or unequal recombination events in the ~75-80 million years since human, for example, shared an ancestor with the prosimian lemur.

The patterns by which SKSD motifs interrupt the SSD repeat domain in primate's closest mammalian relatives, the rodentia and lagomorpha orders, show differences with respect to each other and with primates. The closely related mouse and rat species both have only one SKSD motif found at the amino-terminus of DPP's repeat domain (Figure 3). The two rodents' sequences are very similar with respect to repeat length and lack of SKSD content but a closer look at other imperfections in the SSD motifs illustrates differences in their repeat domain "texture". For example, the motif SSSSS (highlighted in green, Figure 3) whereby the first SSD's aspartic acid group was replaced by a rare third serine, is found eight times in the mouse but not at all in the rat and only once in each primate. Furthermore, the pattern of interspersed dipeptides motifs, predominantly SD and SN (related to the original SSD/SSN motifs by loss of a single serine codon, highlighted in pink, Figure 3) also suggests many different and presumably neutral evolutionary slip replication/recombination events within the ancestor of these two rodents over the last ~15 million years to reach their texturally different but biochemically similar DPP repeat domain.

The other rodent (guinea pig) and the more distantly related lagomorph (rabbit) both lack the SSSSS motif but have many more SKSD motifs throughout their entire repeat domain. This suggests that the common ancestor of rodents and lagomorphs was probably rich in SKSD motifs but the majority of the repeat was lost and replaced with new SKSD-poor repeats by the time of the common ancestor for mouse and rat. (We define a sequence as being "SKSD-rich" if there were, on average, more than 2 SKSD units per 100 amino acids of repeat.) Whether this occurred from a single large recombination event or many smaller events, it demonstrates that over 70 million years, a repeat texture can be highly transformed but the overall serine/aspartic acid-rich nature of the long repeat remains intact. Molecular evolution studies estimate that lagomorphs and rodents separated ~82 MYA; guinea pigs then separated from other rodents (i.e. mouse and rat) about 72 MYA [23]. At first, our data would appear to support these molecular evolution estimates in that both rabbit and guinea pig are SKSD-rich in contrast to mouse and rat which are SKSD-poor. However, the apparent independent loss of the SKSD-rich repeat segments by the primates since their separation from their common ancestor with the rodents/lagomorphs shows that over such time spans, virtually any combination of specific repeat texture motifs may appear, disappear, and/or reappear in the DPP repeat domain.

Most mammals with an intact repeat domain can be considered SKSD-rich. The exceptions include all currently studied primates, elephant, rat, and mouse. Thus, most superorders have both SKSD-rich and SKSD-poor animals suggesting that both sequence types are successful evolutionary pathways and that both textures may appear and disappear within evolutionary lines over time. A closer look at SKSD-rich repeat domains also uncovered textural differences. For example, the abundance and location of the dipeptide motifs (highlighted in pink, Figure 3) are useful to denote the differences and similarities of the repeat domain in these species. As was observed for the SKSD-poor domains, closely related mammals tend to have similar repeat textures. For example, cow and whitetail deer have long SKSD-rich repeats with many dipeptide motifs while their more distant relatives, pig and dolphin are also SKSD-rich but have significantly fewer dipeptide motifs.

The observation in older literature that the amino acid composition of DPP isolated from cow dentin was lysine-rich [10] compared to the DPP of rat [9] and human [37] dentin was the first indication of this evolutionary spectrum. Because both SKSD-rich (cow) and SKSD-poor (rat and human) DPP appear to be well phosphorylated, the presence of positive charges spaced along the repeat domain does not appear to change the outcome of kinase activities. However, verification by a single facility using purified DPP from the teeth of two or more species of each type would be required to definitively conclude that the extent of phosphorylation in both SKSD-rich and poor species is indeed equal.

The overall repeat always remained very acidic due to the presence of many aspartic acids and the presumably phosphorylated serines. Among all the species studied, however, there were some exceptions to the SSD repeat motif that went beyond the introduction of positive amino acids as in SKSD motifs or the loss of a single codon. There were surprisingly few substitutions of the otherwise chemically similar glutamic acid (E) for the aspartic acid (D) even though this substitution would require only a single basepair change. This suggests there was strong evolutionary pressure on retaining D over E amino acids. Because phosphorylation of serines also occurs near glutamic acid residues such as in the SSEE motif found in the early exons of many SIBLINGs, the restriction does not appear to be related to a simple requirement for phosphorylation. (The other acidic SIBLINGs (BSP, DMP1, and OPN) also permit many changes in their protein sequence but appear to have similar restrictions on changes between the two acidic amino acids within their respective acidic domains.)

There was also a curious lack of threonine (T) that could reasonably appear due to a single basepair change of a serine codon (AGC/T to ATC/T) within the repeat domain. Threonine is chemically very similar to serine (S) and can usually be phosphorylated by the same family of kinases so we would have predicted this to be a neutral substitution with respect to most biochemical properties of the phosphorylated DPP. Many species encoded one or two threonines near the 3' end of the repeat domain, but few species had any within the middle of the repeat. Perhaps the efficiency of cooperative phosphorylation along the complete length of the repeat domain by the appropriate kinase(s) could be hindered by intervening threonines, a hypothesis that can be tested in future experiments using DPP sequences that have specific T-for-S substitutions. Curiously, the single basepair change of the negatively charged D to the similarly sized and polar but uncharged asparagine (N) occurred so frequently in mammals that the SSN can be quite common within of the overall "SSD repeat" for most species.

There appears to be a limit on the other amino acids tolerated within the DPP repeat structure. Characteristic of SIBLING genes as a whole and most specifically in DPP, very few hydrophobic amino acids were observed. There were only few exceptions to this rule. For example, one or two isoleucines (I) were found in cow, Asian elephant, and dolphin as well as the single valine (V) in manatee and guinea pig. There were no leucines (L), methionines (M), phenylalanines (F), tryptophans (W) or tyrosines (Y) within the intact repeat domains of any species. Interestingly, mutations resulting from the loss of a single basepair within the DPP repeat changed its normally hydrophilic amino acids (SSD) to predominately hydrophobic amino acids (I, V, and A, for example) causing many cases of human dentinogenesis imperfecta and dentin dysplasia [8,11,12]. Proline was another amino acid highly selected against in the DPP repeat domain with only a single example (SKPD) occurring in armadillo probably within what was previously a SKSD unit (Figure 3). Two cysteines (C) were found within the repeat domains of the platypus DPP although it is not known at this time if intra or intermolecular disulfide bonds form in the dentin or ductal epithelial cell environment in this creature. (All mammals whose DSP domain is available, however, encoded a single cysteine of unknown function within this domain, data not shown.)

Next, we looked for correlations between SSD repeat length and texture with biochemical properties and gross tooth anatomy/function. We could find no obvious correlation between gross tooth phenotype and either the length of the SSD repeat or its relative SKSD content. For example, among carnivores with similar tooth structures, the dog had nearly twice as long a repeat as the cat. Mouse and elephant both had relatively short repeat domains despite the obvious difference in the size of their teeth. Animals that have teeth whose functional surfaces are entirely enclosed in enamel had both long (primates) and short (mouse molars) repeat length as well as SKSD-rich (guinea pig) and SKSD-poor (primates) repeats. Similarly animals with alternating enamel and dentin matrices on their grinding surfaces also had long (whitetail deer) and short (elephant) as well as SKSD-rich (cow) and SKSD-poor (elephant) repeats. While this would suggest that the SSD repeat domain of DPP can perhaps be of any length from ~70 to greater than 230 repeats and be either SKSD-rich or poor and still result in high quality dentin, it remains to be seen if there are some microscopic or biophysical properties we have failed to consider that correlate with specific repeat length and/or specific elements of texture.

It is intriguing that although we have presented evidence that closely related species have significant differences in their specific DPP sequences (as witnessed by the texture of the imperfect repeats), the total length of related species' repeat domains tended to be similar. At least among our limited number of alleles sequenced for each species, examples of difference in lengths of DPP (from their mean value) included: humans and chimpanzees (whose shared ancestor existed ~7 MYA) by ~2%; whitetail deer and cow (~30 MYA) by ~4%; rat and mouse (~15 MYA) by ~5%; and the two elephant species (~7 MYA) by ~7%. The two dolphin species (~20 MYA) were a clear exception to this observation among the mammals completely sequenced to date, differing by ~33% from their mean. There are several distinct selective pressures that one can hypothesize acting upon a gene product's composition over generations. Probably the one most commonly considered is whether the final product (e.g., DPP), at its final location (dentin matrix), performs its function in a way most advantageous to the survival and reproductive success of the species. However, the translation of the DSPP protein itself as well as the addition of post-translational modifications, packaging, and secretion are complex processes that can also place selective pressures on the cells performing these critical functions. Odontoblasts make unusually large amounts of this very acidic protein during dentinogenesis, probably second in abundance only to the two alpha chains of type I collagen. In our laboratory, we have had significant difficulty over expressing even the relatively short mouse *DSPP *using the same viral vector/cell culture system that we have been successful in over expressing the other three acidic SIBLING proteins (BSP, DMP1, and OPN). We have been able to make small amounts of human DSPP lacking nearly all of the repeat domain [38], suggesting that the repeats may cause a significant portion of the problems during biosynthesis/secretion. One example of a possible cellular stress is that the cell must keep DSPP from precipitating or forming a gel in the relatively high calcium ion environment of the rough endoplasmic reticulum (rER). Since the length of the repeat may contribute to such stresses, slip replication or unequal recombination events significantly increasing the size of the repeat domain may be selected against unless the animal co-evolves the mechanisms within the cell machinery to deal with the increased stresses. In the end, some species may, for example, be co-evolving the benefits of a larger repeat (for as yet undefined functions of DPP in the extracellular matrix environment) with the increased stress of actually translating and processing this very acidic phosphoprotein. It is not yet known if animals that make significantly shorter DPP repeat domains, translate more copies of DSPP such that the total content of phosphorylated repeat in their dentin is similar to that found in the dentin of mammals that make DPP with longer repeats.

### Serine, Glycine, and Asparagine-Rich Domains Before and After SSD Repeat

The amino acid sequences between the BMP1 site and beginning of the SSD repeat as well as immediately after the repeat are rich in serine, glycine, and asparagine for all mammals with an intact DPP repeat domain. These areas usually contain several motifs for N-linked oligosaccharide addition (NXS/T), but identifying which of these motifs are actually glycosylated has been solved only for the pig [5]. It is not known at this time if this domain represents a significant retention of sequences due to selective pressures or merely the random drift of this domain with time.

The amino acids near the stop codon (typically SDSNHSTSDD-stop) are interesting because they remain conserved even though a single base pair addition (or 2 bp loss) anywhere within the repeat domain would rapidly result in the appearance of a new stop codon and a foreshortened protein. As discussed above, the repeat lengths can vary within a single species so the shortening of the repeat length by a relatively late (3') +1 frameshift, resulting in a premature stop codon does not appear to be the critical selective force behind the lack of such frameshifts or other introduced nonsense codons. Rather, the non-repetitive amino acid sequence near the stop codon itself appears to be under direct selective pressure. Among the toothed mammals in this study, platypus is the only species that has a short peptide extension (~9 amino acids, highlighted in red in Figure 3) beyond the conserved carboxy-termini. It is interesting to note that we found the carboxy-terminal regions (20-50 amino acids) of each SIBLING to be among the most conserved and therefore most useful motifs for identifying their respective orthologs in more distantly related animals and this observation appears to have held for DSPP.

### Expression of DPP in Toothless Mammals

One way to help distinguish if a protein that is expressed in several tissues is performing a critical function in one particular tissue is to study the gene in species that have lost that tissue/function. For example, Demere *et al*. [39] recently showed that the genes of two enamel-associated proteins, ameloblastin and enamelin, are degraded into pseudogenes in baleen whales, animals that do not make enamel. A short time later, Sire *et al*. [40] noted the loss of the same two enamel genes in the chicken. With such results, one can hypothesize that even if these genes were expressed in other tissues, they cannot serve critical functions outside of the enamel. Although DSPP (DSP + DPP) is the most abundant noncollagenous gene product entrapped within the mineralized matrix of dentin, it is also expressed in metabolically active ductal epithelial cells including kidney, salivary gland, and sweat gland [15-17]. Therefore, one goal was to analyze genetic conservation of the single exon-encoded DPP in species that have lost their ability to make teeth. (DSP is usually encoded within several exons that are not sufficiently conserved to permit similar PCR amplification and sequencing studies at this time.) Genomic DNA was collected from available "toothless" mammals to test the hypothesis that the intact DPP domain of the *DSPP *gene may not be important to species lacking dentin. Baleen whales capture their food using enormous filters made of keratin. As mentioned above, they lack teeth as adults and at least one species has lost two enamel genes, *AMBN *and *ENAM *[39]. We found that one baleen whale (bowhead) had an intact DPP domain that looks very much like other mammals including the relatively closely related dolphins (Figure 3). It turns out, however, that fetuses of at least some baleen whale species develop and then resorb before birth, a dentin-like tooth structure that lacks an enamel covering [39,41]. It is not known if the retention of the DPP domain in these animals is due to selective pressures associated with: 1) the direct production of dentin in their embryonic tooth structures; 2) DPP released during the biosynthesis of these temporary teeth modulating the responses of other nearby tissues (e.g., baleen); or 3) critical expression of DPP in the kidney or some other non-dental tissue.

The platypus, known for its sensitive, rubbery, and "toothless" bill, develops a single, functional if temporary "egg tooth" (apparently used solely for opening its leathery eggshell during hatching) and dentin-containing vestigial molars that are lost by the time they leave their breeding burrow [42,43]. We identified an intact platypus DPP domain with a slightly elongated carboxy-terminus (Figure 3). It is curious that *Dspp*-null mice [13] produce at least minimally functional incisors and molars (teeth that seem likely to function well enough to serve briefly as an "egg tooth" if required) yet the platypus appears to have retained an intact DPP domain. Therefore, either the functional structure of the platypus tooth is more dependent on DPP over evolutionary time periods than is the laboratory *Dspp*-null mouse or this acidic protein is participating in some other, perhaps soft tissue function in this monotreme.

The DPP domain of two genera of anteater (diverging ~13 MYA) that are toothless throughout their entire lifespan were cloned and sequenced. As seen in the translated sequences of Figure 3, each species independently introduced a stop codon near the beginning of DPP. The giant anteater had a stop codon three amino acids after the BMP1 motif while the Tamandua exhibited two single basepair deletions leading to two successive frameshifts about where the repeat would normally begin. The first frameshift caused the production of ~30 alternative amino acids (12 of which are hydrophobic) and then the next frameshift caused the reading frame to shift into one that had many stop codons. (A substantial amount of the remnants of the repeat domains can be observed by translating all three reading frames beyond the stop codons.) It is clear that if DSPP is translated, it will lack a discernable DPP domain. (We did not, however, sequence the more poorly conserved DSP-encoding exons which could also have deleterious mutations in these two animals. If this were the case, then the common ancestor of the two anteaters may have already lost their functional DSPP and the mutations we observed in DPP may have occurred later. The future publication of the entire DSPP-encoding domain of either anteater genome will, of course, shed light on this interesting point.) The only other Xenarthra we successfully sequenced was the armadillo, an animal with crude, peg-like teeth that contain dentin but little or no enamel covering. This only living Cingulata shared a common ancestor with the Pilosa order (anteaters and sloths) about 9 million years prior to the divergence of the two anteater genera and had an intact DPP domain (Figure 3). In summary, it appears that the two truly toothless animals sequenced to date have independently lost their functional DPP suggesting that DPP (but not necessarily DSP) may perform a critical function only in dentin. The extremely narrow diet and focused lifestyle of both anteaters, however, does raise an interesting question as to whether or not the possible functions of DPP in their kidneys and specialized salivary glands, for example, are not as important to the physiology of anteaters as they may be for many other mammals. (Indeed, it is not known if DSPP is expressed in ductal epithelial tissues in mammals other than primates and rodents.) Therefore, the analyses of the *DSPP *gene from other "toothless" animals would be helpful in determining the validity of the hypothesis that DPP may be required only for dentin.

Unfortunately our attempts to PCR amplify and sequence the DPP domain was unsuccessful for the only other truly toothless animal for which we could obtain genomic DNA, the pangolin (*Manis tricuspis*). The lack of a PCR amplicon at first suggests that this mammal may lack a functional DPP domain, but as discussed earlier, we were also unsuccessful at amplifying this domain from several other species with fully functional teeth. We expect that in the next few years, the pangolin genome project conducted by The Genome Sequencing Center at Washington University in St. Louis School of Medicine, will elucidate an entire DPP domain (or its remnant) or, as was the case for several mammals in this report, at least the 5' and 3' ends thereby permitting the design of new pangolin-specific PCR primers.

### DSPP Gene Analysis of Non-Mammalian Species

**Figure 4:**
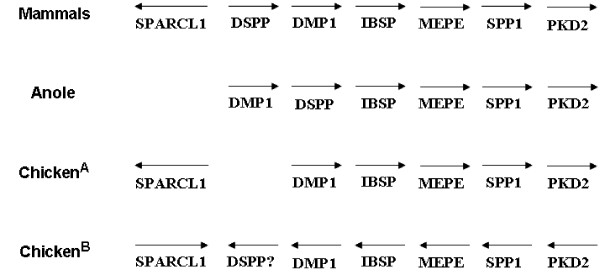
**Order and transcription direction (arrows) of SIBLING genes plus adjacent *PKD2 *and *SPARCL1 *genes in mammals, green anole, and chicken**. Note that the order of the *DSPP *and *DMP1*-like genes are reversed between mammals and the anole without changing their direction of transcription relative to the surrounding genes. Row labeled Chicken^A ^is our interpretation of the relative directions of all six genes' transcription as based on the version 2.1 chicken genome from the Genome Sequencing Center at the Washington University School of Medicine (St. Louis, MO) as compared to the interpretation by Sire et al. [40] (Chicken^B^).

The SIBLING gene clusters in Figure 4 illustrate the order and transcription direction of the five SIBLING genes as seen in all mammals completed to date. Except for a few characteristic motifs, each of the SIBLING orthologs undergoes a significant amount of change. Therefore, the order of the genes is also highly useful information in the search for specific orthologs in non-mammalian species. Fortunately, the SIBLING genes are also often flanked on one side by the conserved *SPARCL1 *(known in some species as *HEVIN *or *MAST9*) and on the other side by the conserved *PKD2 *gene, making it possible to direct the search for the *DSPP *gene within the gene cluster in non-mammalian species by association with one or both orthologs of these two flanking genes. The data obtained from the genome project for the reptile, green anole (*Anolis carolinensis*, Broad Institute AnoCar-1.0), was sufficient to assign each gene in the SIBLING gene cluster by analogy to motifs found in their mammalian orthologs (Figure 4). Three reports briefly note the presence of a *DSPP*-like gene in the lizard genome through this same approach [2,40]; although the stated gene order of *DSPP *and *DMP1 *within the proposed stem amniote's SIBLING gene cluster (acidic SCPP) is not the same as we observed [44]. Our analysis of the contiguous sequence scaffold shows the order and orientation of the five SIBLING genes as well as *PKD2 *are the same as those observed in mammals except the gene most resembling *DMP1 *is located at the 5' end of the SIBLING cluster, a location held by *DSPP *in all mammals to date. The anole's *DMP1 *has the classic SSEE phosphorylation motif found early in the translated sequence, SGDD glycosaminoglycan-attachment motif, MQGDD BMP1-cleavage motif, and the characteristic carboxyl-terminus (HNKPXXDXDDNDCQDGY*). (Although the anole genome project is unfinished, no obvious *SPARCL1 *gene was observed within the scaffolds immediately 5' to the SIBLING gene cluster.)

The anole *DSPP *gene was defined by first locating the *DMP1 *gene homolog and then manually searching for a nearby large, serine/aspartate-rich open reading frame within a contiguous sequence scaffold. A 1722 bp open reading frame was found 3' to *DMP1 *and 5' to the anole *IBSP *ortholog (Figure 4). This large open reading frame encoded a classic BMP1 cleavage site (MQGDD) followed 16 amino acids later by a remnant of the RGD motif (RGQ), a >250 amino acid serine/aspartate-rich domain, and finally the *DSPP*-like SNNSTSDE motif near the slightly extended carboxy-terminus (somewhat reminiscent of the platypus's extended DPP carboxy-terminus, Figure 3). A short exon 5' to this large open reading frame encoded a leader sequence that upon cleavage by the signal peptidase would leave a proline in position number two of the mature protein. When this exon is spliced to the large open reading frame, the translated protein becomes linked to the first amino acid of next exon, a leucine, resulting in a mature protein starting with serine-proline-leucine. As published previously, human *DSPP *gene exon splicing results in a mature protein that starts with a similar isoleucine-proline-valine tripeptide, where the proline-hydrophobic amino acid dipeptide portion appears to be necessary for correct processing of DSPP. A variety of mutations that disrupt the biochemical properties of these two amino acids resulted in the nonsyndromic, dominant-negative dentin disorder, dentinogenesis imperfecta (DGI) [8]. The sequences of the splice sites for these two reptilian exons fit the classic GTAAG and CT-rich domains, suggesting that this splice event could occur. Furthermore, both splice sites have significantly positive SplicePort http://spliceport.cs.umd.edu/ sensitivity scores of 0.5 for the donor site of the leading exon and 0.8 for the acceptor site of the large repeat encoding exon, respectively. No other obvious open reading frames or splice donor/acceptor pairs were observed in the ~2 kb of sequence between these two coding exons suggesting that green anole *DSPP *may have only two coding exons with the second one containing both DSP and DPP-like domains separated by BMP1 at the conserved MQGDD motif.

We observed a reversal in the order of the *DMP1 *and *DSPP *genes in this reptilian genome but not a reversal of their transcription direction relative to the remaining SIBLING genes and *PKD2 *suggesting that a simple local inversion of the two genes does not explain the changes. Although a more complex gene reorganization explanation is possible, an interesting alternative possibility exists. It is possible that a duplication of an ancestral *DMP1*-like gene separately gave rise to both modern *DMP1 *and *DSPP*. In this hypothesis, the 5' copy of the reptilian line's primordial *DMP1 *retained its original *DMP1*-like properties (i.e. BMP1-cleavage, glycosaminoglycan-attachment, integrin-binding RGD, and carboxy-terminal motifs) while a repetitive SSD unit was expanded in the 3' copy to give rise to the *DSPP*-like gene. In the mammalian line, a similar SSD expansion occurred in the 5' copy of the ancestral *DMP1*. These separate but similar expansions of the phosphorylation sites on different copies of an ancestral *DMP1 *gene may have resulted in the apparent reversal of the gene order between mammals and reptiles. Detailed comparison of the sequences encoding the reptile's serine/aspartate-rich DPP domain to that found in all mammals offers support for this hypothesis. The repeat domain for the anole DPP is encoded by both TCN-type (the bold **S**'s in anole, Figure 3) and AGC/T-type serine codons throughout its entire length. This is in stark contrast to the mammalian repeat in which all serines are encoded solely by AGC/T-type codons. (Both TCN and AGC/T-type serines are present 5' to the repeat domain in mammals.) Sequential single basepair mutations are highly unlikely to explain the large number of differences between the patterns of serine codon usage in lizard and mammalian DPP repeat domains. It is more likely that expansion of different nominal SSD repeat units from the ancestral *DMP1 *account for the biochemically similar but distinctly differently-derived repeat structures. In addition, the anole DPP domain has independently acquired positively charged arginine amino acids (blue highlighting in anole, Figure 3) spaced along the repeat length in a pattern similar to the positively charged lysines (as SKSD motifs) in most mammals. Thus, both lizard and mammalian lines of evolution appear to have independently developed DPP-like domains containing extensive phosphorylated serine motifs interspaced with positively charged amino acids.

The final species analyzed was chicken (*Gallus gallus*), a toothless species that diverged from the common bird-reptile ancestor ~210 MYA. Using the same approach as performed for the anole, the 43.5 kb chicken *SPARCL1*-SIBLING-*PKD2 *domain from the chicken genome project (v2.1, Washington School of Medicine in St. Louis, accessed through the UCSC Genome Browser) was scanned for open reading frames corresponding to the five SIBLING genes. Four chicken SIBLINGs (*SPP1*, *OC116 *or *MEPE*, *IBSP*, and *DMP1*) on chromosome 4 were in the same order and transcription orientation as their mammalian orthologs. The flanking genes, *SPARCL1 *and *PKD2*, were also in the same relative locations and orientations as their orthologous human genes. In this sense, our analysis of the database is strikingly different than the recent report by Sire *et al*. [40], which concluded that although the order of the chicken SIBLINGs and two flanking genes were the same as in human, the transcription direction for each SIBLING ortholog, as well as both flanking genes, were individually inverted (Figure 4). In agreement with Sire *et al*. [40], we could find no large open reading frames or significant remnants of *DSPP *in its expected location between *SPARCL1 *and *DMP1 *or 3' to *DMP1 *as was the location of the reptilian *DSPP*. The loss of *DSPP *(presumably the reptilian form) in the evolution of the toothless chicken supports the hypothesis that DPP is important in dentin biosynthesis/function.

## Conclusions

Our analysis would suggest that the most recent addition to the SIBLING gene family, *DSPP*, arose from an ancestral *DMP1 *gene duplication and subsequent evolution into a new gene that retained some of the DMP1 motifs (for example, BMP1 cleavage and RGD) while gaining some novel domains (i.e. a long and repetitive phosphorylation domain). It is possible that different phosphorylation motifs (using both AGC/T and TCN-encoded serines in reptiles and exclusively AGC/T-encoded serines in all mammals) were independently expanded many times to give rise to biochemically similar but distinct phosphorylated serine/aspartate-rich repeat regions that are a hallmark of DPP. The integrin-binding RGD motif was found to be independently lost in at least one animal in most branches of the mammalian phylogenic tree. However because one or more RGD motifs appeared at different locations in at least two mammals that lost its original RGD domain, we urge caution against firm conclusions that binding to cell surface receptors by DSPP is evolutionarily neutral in all species. The conservation of DPP and it's repetitive domain in all toothed mammals, including two species that are toothless as adults (platypus and baleen whale), as well as the subsequent loss of DPP in two truly "toothless" mammals (two genera of anteaters) and chicken, does suggests that the physiological relevance of DPP may be limited to the formation or function of dentin or dentin-associated structures, but additional toothless mammals (e.g. pangolins) need to be sequenced to strengthen this hypothesis.

## Authors' contributions

DM participated in the cloning, molecular genetic analysis, sequence alignments, and drafted the manuscript. LF designed the project, participated in the molecular genetic analysis, as well as contributed to the writing of the manuscript. Both authors have read and approved the final manuscript.

## Supplementary Material

Additional file 1 Optimized PCR Methods Click here for file

Additional file 2 Optimized Sequencing Primers Click here for file

Additional file 3 The DPP sequences of 26 mammalian species and green anole. Larger Font version of Figure 3. Click here for file
