# Supplementary material for: Molecular evolution of dentin phosphoprotein among toothed and toothless animals
Source: BMC Evol Biol. 2009 Dec 23;9:299. doi: 10.1186/1471-2148-9-299 (PMC2803795; doi:10.1186/1471-2148-9-299)
Supplement: Additional file 1 — Optimized PCR Methods [file 1471-2148-9-299-S1.PDF]

Optimized PCR Methods

Species	Forward Primer	Reverse Primer	Cycle number	Anneal Temp.	Extension Time
Standard DPP Conditions	5´AGTCCATGCAAGGA GATGATCC3´	5´CTAATCATCACTG GTTGAGTGG3´	35	55°C	3 min
Opossum	5´ARTCCATGCAAGGA GATGAYCC3´	5´GTTCTAATCATCA CTGGTTGAGTG3´	35	52°C	3 min
Giant anteater	Same as opossum	Same as opossum	40	52.5°C	3 min
Armadillo	5´GAATCCATGCAAGG AGATGATCC3´	5´CTAATCATCACTT GTCGAATGATTAC3´	35	55°C	3 min
Platypus	5´GATGGATATGATGA GAGCTATGATTTC3´	5´GGTACCTTGTGGT ATCACTTCGAG3´	35	55°C	6 min
Whale	Same as standard	5´CTAATCATCACTG GTTGAGTG3´	35	50°C	3 min
Bottle nose dolphin	Same as standard	Same as whale	35	50°C	3 min
Short-beaked common dolphin	Same as standard	Same as whale	35	50°C	3 min
Manatee	Same as standard	Same as whale	35	52°C	3 min

A “R” in the primer indicates a degenerate base location where an adenine or guanine was used. A “Y” in the primer indicates a degenerate base location where a cytosine or thymine was used.
