# Supplementary material for: Molecular evolution of dentin phosphoprotein among toothed and toothless animals
Source: BMC Evol Biol. 2009 Dec 23;9:299. doi: 10.1186/1471-2148-9-299 (PMC2803795; doi:10.1186/1471-2148-9-299)
Supplement: Additional file 2 — Optimized Sequencing Primers [file 1471-2148-9-299-S2.PDF]

Optimized Sequencing Primers

Species	Sequencing Primers
Dasypus novemcinctus (Armadillo)	5´GACAGTAAATCAGACAGCAGG3´
Bos taurus (Cow)	5´GGCAATGATGACAATGGAGG3´<br>5´CAAATCAGGCAACAGCAAAGATAAA3´<br>5´CAGTGACAGTAAATCAGACAAC3´<br>5´GCTGTCACTGTCAGATTTAG3´
Odocoileus virginianus (Deer)	5´GAATGGAAAATCAGGCAGCAC3´<br>5´GCTGTTGCTGTCACTCCT3´
Tursiops truncatus (Dolphin, Bottlenose)	5´GTGATAAGAATGGAAAATCAGGC3´<br>5´GCTGTTGCTGTCACTCCT3´
Gorilla gorilla (Gorilla)	5´CAGACAGTGGCAAAGGTAAA3´
Oryctolagus cuniculus (Rabbit)	5´CAGTGACAGCACATCACACAC3´
Ateles geoffroyi (Spider Monkey)	5´CAAGTCAGACAGCAGCAAA3´
Balaena mysticetus (Whale, Bowhead (baleen))	5´GAATGGAAAATCAGGCAGCAC3´
