# Supplementary material for: Molecular evolution of dentin phosphoprotein among toothed and toothless animals
Source: BMC Evol Biol. 2009 Dec 23;9:299. doi: 10.1186/1471-2148-9-299 (PMC2803795; doi:10.1186/1471-2148-9-299)
Supplement: Additional file 3 — The DPP sequences of 26 mammalian species and green anole. Larger Font version of Figure 3. [file 1471-2148-9-299-S3.DOC]

**Bowhead (Baleen) Whale**
MQGDDPNSSGESNGSDDTNSEGNNSHSSRGDASYNSDESSDNGNDSDSKGGEEDDSDNTSDANDSGSDGNGNNGSDKSGKSGSTKDKSDSSDSSDSSDSKSDSSDSSNSSNSSESSDSSDSSDSSDSSDSSSGSKSDSSDSSNSSDSKSDSDSSESSDNSDSSDSSDSSDSSDSKSDSSDSSDSSGSDSKSDSDSSDSSDSDSKSDSDSSDSSDSKSDSSDSSDSSDSDSKSDSDSSDSSDSSDSSDSKSDSSDSSDSKSDSSDSSDSSDSDSSDSDSSDSDSSDSSDSDSSDSSDSSDSDSSDSSDSDSSDSSDSSDSDSSDSASDSGDESDSKSKSGNGNNNGGGSDSDSDSEGSDSNHSTSDD*

**Short-Beaked Dolphin**

MQGDDPNSSDESNGSDDTNSEGDNNHSSQGDASYNSDESSDNGNDSDSKGGEEGDSDNTSDANDSGGDGNGDIGSDKNGKSGSTKDNSDSSDSSDSSDSSDSSDSSDSKSDSSDSSNSINSSDSSDSSDSSDSSDSSDSSNGSNSSESSDSSDSSDSSDSSDSSDSSDSSDSSDSSNGSNSSESSDSSDSSDSSDSSDSSDSSDSSDSSSGSKSDSSDNSDSSDSSNSSDSKSDSSDSSNSINSSESSDSSDSSDSSDSSDSSESSDSSDSSGSSKSDSSDSSNSSDSKSDSSDSSDSSDSSDSSDSSDSSDSSDSGDSKSDSSDSSDSSESDSKSDSDSSNSSDSKSDSSDSSDSSDSSDSSDSQSDSSDSSDSSDSDSSDSDSSDSDSSDSDSSDRSDSNSSDSNSSDSDSSDSASDSGDESDSKSKSGNGDNNGGGSDSDSDSKGSDSNHSTSDD*

**Bottlenose Dolphin**

MQGDDPNSSDESNGSDDTNSEGDNNHSSRGDASYNSDESSDNGNDSDSKGGEEGDSDNTSDANDSGGDGNGDMGSDKNGKSGSTKDNSDSSDSSDSSDSSDSSNSSESSDSSDSSDSSSGSKSDSSDSSDNSDSSDSSNSSDSKSDSSDSSNSINSSESSDSSDSSDSSDSSDSSDSSNSSDSSDSSDSSDSSDSSNSSESSDSSDSSDSSSSSKSDSSDSSNSSDSKSDSSESSDSSDSSDSSDSGDSKSDSSDSSDSSESDSKSDSDSSNSSDSKSDSSDSSNSINSSESSDSSDSSDSSDSSDSSDSSNSSDSSDSSDSSDSSDSSNSSESSDSSDSSDSSSSSKSDSSDSSNSSDSKSDSSESSDSSDSSDSSDSGDSKSDSSDSSDSSESDSKSDSDSSNSSDSKSDSSDSSDSRDSSDSSDSKSDSSDSSESSDSSNSSDSSDSSDSSDSSDSKSDSSDSSESSDSSDSSDSSDSGDSKSDSSDSSDSSESGDSKSDSSDSSDSSESDSKSDSDSSNSSDSKSDSSDSSDSSDSSDSKSDSSDSSDSKSDSSDSSDSSDSDSSDSDSSDSDSSDSDRSDSNSSDSDSSDSASDSGDESDSKSKSGKGDNNGGGGDSDSDSKGSDSNHSTSDD*

**Whitetail Deer**

MQGDDPNSSDDANSEGDNNHSSRGDTSYNSDESNDHGNDSGSKGDDDGSDNTSDANDSGSDGNGNNGDDDNGKSGNSKDKSDSSDSSDSSESDSKSDSDDSDSDSSDSSDSSESDSKSDSDDSDSSDSSDSSDSSDSSDSSESDSKSDSDDSDSSDSSDSSDSSDSSDSSDSDSKSDSDSSDSSDSSDSSDSSESDSKSDNDSSDSDSSDSSDSDSSDSSDSDSSDNSDSSDSKSDSSDSSDSDSKSDSSDSSDSSDSSDSSDSDSKSDSDSSDSSDSSDSKSDSSDSSNSDSKSDSDSSDSSDSSDSSDSDSKSDSSDSSDSDSSDSSDSSDSDSKSDSDSSNSSDSSDSSDSDSKSDSSDSSDSDSKSDRDSSDSSDSDSKSDSDSSDSSDSSDSSDSSDSSDSDSSDSSDSDSKSDSDSSDSSDSDSSDSSDSDSKSDSDSSDSDSSDSSDSSDSDSSDSSDSDSKSDSDSSDSSDSDSSDSSDSDSKSDSDSSDSDSSDSSDSSDSDSSDSSDSDSKSDSDSSDSSDSSDSSDSSDSDSSDSSDSDSSDSDSSDSSDSSDSDSKSDSDSSDSSDSSDSSDSSDSSDSDSSDSSDSDSKSDSDSSDSNSDSSDSSDSSDSDSKSDSDSSDSSDSSDSSDSDSKSDSDSSDSDSSDSDSSDSSNSDSSDSSDSDSKSDSDSSDSSDKSDSKTKSGKGNNNGSSSDSDSDSEGSDSNHSTSDD*

**Cow**

MQGDDPNSSDESNGSDDANSEGDNNHSSRGDASYNSDESNDNGNDSGSKGDDDGSDNTSDANDNGSDGNDDNGGDDSGKSGNSKDKSDSSDSSDSSDSDSSDSSESDSKSDSDSSDSSDSDSKSDNDSSDSSDSDSKSDSDSSDSSDSKSDSSDSSDSDSSDSSDSKSDSSDSSDSSDSDSSDSSDSSDSSDSSDSDSKSDSDSSDSSDSKSDSSDSSDSDSSDSSDSKSDSSDSSDSSDSDSSDSSDSSDSDSSDSSDSDSKSDSDSSDSSDSKSDSSDSSDSDSSDSKSDSSDSSDSDSKSDSDSSDSSDSKSDSSDSSDSSDSDSSDSSDSSDSSDSKSDSSDSSDSSDSDSSDSDSSDSSDSSDSDSKSDSDSSDSSDSKSDSSDSSDSSNSSDSSDSSDSSDSSDSSDSSDSKSDSSDSSDSSDSSNSSDSDSKSDSDSSDSSDSSDSSDSSDSKSDSSDSSDSDSSDSSDSSDSSDSSDSSNSSDSDSKSDSDSSDSSDSSDSSDSSDSKSDSSDSSDSDSSDSSDSSDSSDSSDSKSDSSDSSDSSDSKSDSSDSSDSDSKSDSDSSDSSDSSDSDSSDSSDSSDSDSSDSSDSDSKSDSDSSDSSDSSDSSDSSDSDTKSDSDSSDSSDSDSSDSSDSSDSSDSSDSDSSNSSDSDSKSDSDSSDSSDSDSSDSSDSDSSDSSDSDSSDSSDSNDSNTKSDSDSSDSSDSSDSKSKSGNGNHNGGSSDSDSDSEGSDSNHSTQ*

**Pig**

MQGDDPNSSEESNGSDDANSEGDNNHNSRGDTSYNSDESDDNGNDSDSKEEAEEDNTSDANDSDSDGNGDNGSDDSGKSGSSKAESESSQSSESSESDSSESNDSSESSDSSDSKSDSSESSDSSDSSDSSDSSDSSDSKSDSSDSSDSSDSSDRSDSSDSSDSSDSKSDSSDSSDSKSDSSDSSDSSDSSDSSDSSDSSDSKSDSSDSSDSSESKSDSSDSSDSSDSKSDSSDSSDSKSDSSDSSDSSDSKSDSSDSSDSSDSKSDSSDSSDSKSDSSDSSDSSDSSDSSDSSDSKSDSSDSSDSKSDSSDSSDSSDSNDSKSDSSNSSDSSDSSDSSDSSDSKSDSSDSSDSKSDSSDSSDSSDSSDSKSDSDSSDSSDSSDSSDSSDSSDSSDSKSDSSDSSDSKSDSSDSSDSSDSDSSDSKSDSSDSSDSKSDSDSSDSSDSSDSSDSDSSDSKSDSSDSSDSSDSSDSSDSSDSSDNDSSDSSDSDSSDDSNSSDSSDSDSSDGSDSSDSSDSDSNDSSDSSDSSDSSDSASDSSDEGDSKSKAGNGDNNGSDSDSSSDSEGSDSNHSTSDD*

**Cat**

MQGDDPNSSDESNDNDDVNSEGDNNSDSRGDPGYDSDESKDNGNDSDSNGGDDDDSDSTSDANDSDSNGNGNNGSNDNGKPDSSKDKSDSSDSSDSSDSSDSSDSSDSSDSSDSSDSSDSNSSDSSDSSDSDSSDSSDSSDSKSDSSDSSDSSDSSDSSDSSDSSDSKSDSSDSSDSSDSSDSSDSSDSSDSKSDSSDSSDSSDSSDSSDSSDSSDSKSDSSDSSDSSDSSDSKSDSSDSSDSSDSSDSKSDGSDSSDSSDSSDSSDSSDSSDSSDSKSDSSDSSDSDSKSDSSDSGNSKSDSSDSSDSDSKSDSSDSSNSKSDSDSSDSSNSDSSDSSDSSDSENSDSSDSSDSDNSDSSDSSDSNSSDSSDSDSSDSSDSDSSDSASDSSDESHSKSKAGNGNNGGDSDSDSEGSDSNHSTSDD*

**Dog**

MQGDDPNISDESNGNDDADSEGDNNSDSQGDNGYNSDESKDNGNDSDSNGGGDDDSDSTSDANESDSNGNGNNGSDDNGKSESSKDKSDRSDSSDSSDSSDSSDSSDSKSDSSDSSDSSNSSDSSDSKSDSDSSDSDSSDSDSKSDSSDSSDSSDSSDSSDSSDSSDSSDSDSKSDSSDSSDSSDSSDSSNSDSSDSSDSSDSSDSSDSSNSDSSDSSDSSDSSDSDSKSDSSDSSDSSDSSDSSDSSDSDSKSDSSDSSDSSDSSDSSDSSNSDSSDSSDSSDSDSKSDSSDSSDSSDSSNSDSSDSSDSSDSDSDSKSDSSDSSDSSDSSDSSNSDSDSKSDSSESSDNSNSDSNSKSDSSDSSNSKSDSSDSSDSSNSKSDSDSSDSSDSDSKSDSSDSSSSSSKSDSSDSSKSDSDSSDSSDSSDSSDSSDSDSSDSSDSSNSSDSSDSDSSDSSDSSDSSDSSDSSDSSNSSDSSNSSDSSNSSDSSDSDSSDSSDSDSSDSDSSNSSDSSESDSSDSDSDSSNSSDSSDSDSSDSSDSDSSDSSNSSDSDSSDSDSDSSNSSDSSDSDSSDSDSDSDSDSNSNSSDSSDSNSDSSDSDSSDSSDSASDNDESHSKTNSGNGKNGNDSESDSEDSDSNHSTSDD*

**Mouse**

MQGDDPKSSDESNGSDESDTNSESANESGSRGDASYTSDESSDDDNDSDSHAGEDDSSDDSSDTDDSDSNGDGDSDSNGDGDSESEDKDESDSSDHDNSSDSESKSDSSDSSDDSSDSSDSSDSSDSSDSSDSSDSSDSSDSSDSNSSSDSSDSSSSSDSSDSSDSSDSSDSSDSSDSSDSSDSSDSSDSSDSSDSSDSSDSSSSSDSSDSSSSSDSSDSSDSSDSSDSSDSSDSSSSDSSSSSNSSDSSDSSDSSSSSDSSNSSDSSDSSDSSDSSDSSDSSNSSDSSDSSSSSDSSDSSSSSDSSDSSDSSDSSESSESSDSSNSSDSSDSSDSSDSSDSSDSSDSSDSSDSSNSSDSSDSSDSSDSSDSSNSSDSSDSSDSSDSSDSSDSSDSSDSSDSSDSSDSSDSSDSSDSSDSSDSSDSSDSSDSSDSSDSSDSSNSSDSSDSDSKDSSSDSSDGDSKSGNGNSDSNSDSNSDSDSDSEGSDSNHSTSDD*

**Rat**

MQGDDPNSSDESNGSDGSDDANSESANENGNHGDASYTSDESSDNGGDSDSYAGEDDSSDDTSDTDDSDSNGDDDSESEDKDESDNSNHDNDSDSESKSDSSDSDSDSSDSSDSSDSSDSSDSSDSSESSDSSDSSDSSDSSDSSDSSDSSDSSDSSDSDSNDSSDSSDSSDSSDSSDSSESSDSSDSSDSSDSSESSESSESSDSSDSSDSSDSSDSSESSESSDSSDSSDSSDSDSSDSSDSSDSSDSSDSSDSSDSSDSSDSSDSSDSDSSDSDSSDSSDSSDSSDSSDSSDSSDSSDSSNSDSSDSSDSSDSSDSSDSSDSSDSSDSSDSSDSSDSSDSSDSSDSSDSSDSSDSSDSSDSSDSSDSSDSSDSSDSSDSSDSNSSDSSDSSDSSDSSDSSDSSDSSDSSDSSDSSDSSNSSDSSDSSDGDSSDGDSSDSDSSDSDSSNSSDSDSSDSSDSSSSDSSDSDSDSKDSTSDSSDDDSKSGNGNSDSDSDSEGSDSNHSTSDD*

**Guinea pig**

MQGDDPHSSDESHGSDGTDSEGDNGNSSRGDVTYNSNESNDSGDNSDSDGEDDGDTDSTPDANDGDGDGDSEREGSAEAGGSDSDENGDSKADSSDSDDNDSNSDNSDSSDSGDSDSSDSKSDSNDGESSVNDSSDSDSKSESSDGDSSDGDSSDGDSSDSSDSDSSDGDSKSDSSDGDSSDSSDSDSKSDSSDSSDGDGSDSKSDSSDGDSSDSSDSDSKSDSSDSSDSDSSDSSESDSKSDSSDGDSSDSSDSDSKSDSSDSSDGDGSDSKSDSSDGDSSDSDSKSDSSDSSDSDSSDSSDSDSKSDSSDSDSSDSSDGDSKSDSNDGDSSDSSDSDSSDSDSQSDSSDGDSSDGGSSDSKSDSSDGDSSDDDSKSNSSESDSKSDSSDGDSSDSSDRDSDSSDSKSDSGDGDSSDSSDSDSKSDSSDGDSSDSDSKSESGDGDSSDSDRKSESSDGDSSDSDSKSESSDGDSSDSDNKSESSDGDSRDGDSSDSDSSDSSDSDSSDSSDSSDSSESDSKSDSSDGDSSDSDSSDSSDSDSSDSSDSSESDSKSDSSDGDSSDSSDSSDRDSSDCSNSSGSNDGSDGRDSRDNNTSDSSDENDTQSKSGDNDNGSDSDSDSEGSDSNHSTSDD*

**Rabbit**

MQGDDPNSSDESNGSDDANSEGDNDNNSQGDTSNNFDESQNKDNDSDSQGEGDDGNSDSTSHTNDSDSNGNGNDDDDSSDSSDGSETGSKSDSTDSSDNSDGTDSSDNKSDSSDSSDNDSKSDSSDSDSSDSSDSDSSDSSDSSDSSDSDSKSDSSDSSDSSDSSDSDSDSSDSDSKSDSSDGSDSSDSSDSDSDSSDSDSSDSDSKSDSSDSGDSSDSSDSSDSDSKSDSSDSSDSSNSSDSDSKSDSSDSGDSSDSSDSSDSSDSDSSDSDSKSDSSDSSDSDSKSDSSNSDSSDSSDSDSSDSDSKSDSSDSSDSDSKSDSSNSDSSDSSDSDSKSDSSDSGDSSDSSDSSDSSDSDSSDSDSKSDSSDSSDSDNDSSDSDSKSDSSDSSDSSDSSDSSDSASSDSDSKSDSSDSSDSDSKSDSSNSDSSDSSDSSDSDSSDSNNSSDSDSSDSNSSDSDSNDSSDSSDSSDSDSKSDSSDSSDSDSSDSDSKSDSSDSSDSDSKSDSSDSSDSSDSSDSDSSDSDSKSDSSDSSDSDSKSDSSDSSDSSDSSDSSDSDSKSDSSDSSDSDSSDSSDSDSSDSDSSDSSNSSDSDSSDSDSNDSSDSTSDSSDDSDSQSKPGNGNNSDSDSESESEGSDSNHSTSDD*

**Lemur**

MQGDDPNSSDESNGSDDANSESDNDSSSPGDASDNSDESKDNGNDSNSKEGSDDGDSTSDSNDSDSNGNGNNGSDDNDKSDSSKGKSNNSDSSDSSDSSDSSDSSDSSDSSDSSDSSDSSDSSDSSDSSDSSDSSDSSDSSDSSDSSDSSDSSDSSDSSDSSDSSDSSDSSDSSDSSDSSDSSDSSDSSDSSDSSDSSDSNSSDSNSSDSSDSSDSSDSSDSSDSSDSDSDSSDSNDSSDSDSKSNSSDSSDSDSKSDSSNSSDSKSDSSDSDSKSDSSDSSDSKSDSSDSSNSSDSSDSSDSSDSSDSSDSSDSSDSSDSSDSDSSDSDNKSNSSDSSDSDSKSDSSNSNDSKSDSSDSSNSSDSSDSSDSSDSSDSSNSSSSSDSSDSSNSDSSDSSDSSNSDSSDSSDSSDSSDSSDSSDSSHSSDSSDSSDSDSSDSDSSDSSDSSNSSDSSDSSDSSDSSNSSNSSDSSDSSDSSDSSDSSDSSDSSDSSDSSNSSDSSDSSDSSDSSDSSDSSDSSDSSDSSDSSDSSDSSDSSDSSDSSDSSDSSDSSDSSDSSDSSDSSDSSDSSDSSDSSDSSDSTSESSDESDSQSKSGNGNNNGSGSDSDSDSEGSDSNHSTSDD*

**Spider Monkey**

MQGDDPNSSDESNGNDDANSESDNNSSSRGDASYNSDESKDNGNGSDSKGAEDDDSDSTSDTNNSDSNGNGNNGNDDNDKSDSGKGKSDSSDSDSSDSSNSSDSSESSDSDSSDSNSSSDSSESSDSDSSDSNSSSDSDSSDSDSSDSNSSSDSSNSDSDSSDSSDSDSSDSSNSSDSSDSSDSDSSDSNSSSDSSDSSDSDSSDSSDSDSSDSSNSSDSSDSSDSSDSSDSSDSSESKSDSSKSDSNSSDSDSKSDSSDSNSSDSSDNSSDSNSSDSSDSSNSSNSSDSDSSDSSDSSSSSDSSNSSDSSDSSDSSNSDSSNSSESSDSSDSSDSDSSDSSDSSNSSDSSNSDSSDSSDSNDSSNSSDSSDSSNSSDSSDSSDSSDSSDSSDSSNSSDSSDSSDSSDSSDSNDSSNSSDSSDSSNSSESSDSSDSSDSSDSSDSSDSDSSNSSDSSNSDSSDSSDSSNSSDSSDSSDSSDSSDSDSNDSSNSSDSNSSDSNDSSNSSDSSDSSNSSDSSDSSDSSDSSDSSDSSDSSDSNSSNSSDSSNSSDSSNSSDSSNSSDSSDSSNSSNSSDSSNSSDSSDSSNSSDSSDSSDSSNSSDSSNSSDSSNSSDSSDSSNSSNSSDSSDSSDSSDSSDSSDSSDSSDSSDSSDSSESSDSDSSDSSDSSNSSDSSDSSDSSDSSDSSDSSDSSDSSDSSDSSNSSDSSDSSDSSDSSDSSDSNESSDSSDSSDSSDSSDSSDSSDSSDSSDSSDSSDSSNSSDSSDSDSTSDSNDESDSQSKSGNGNNNGSDSDSEGSDSNHSTQ*

**Rhesus Macaque**

MQGDDPNSSDESNGNDDANSESDNDSSSRGDASYNSDESKDNGNGSHSKGEEDDDSDSTSDTNNSDSNGNGNNGNDDNDKSDGGKGKSDSSDSSDSSDSDSSDSSNSSDSSDSSDSDSSDSDSSDSNSSSDSSDSSDSDSSDSNSSSDSSDSSDSDSSDSNSSSDSDSSDSDSSDSNSSSDSSDSSDSDSSDSNSSSDSSDSSDSDSSDSNSSSDSSDSSDSDSSDSNSSSDSSDSSDSSDSSDSSDSSDSDSSDSSDSDSSDSSNSSDSSDSNSSDSSDSSDSSNSSDSSDSSNSDSSDSSDNSDSKSDSNKSDSSDSDSKSDSSDSNSSDSSDNSDSSDSSNSSDSSDSSDSSDSESSSSSDSSNSSDSSDSSDSSNSSDHSDSSDSSDSSDNSNSSDSSDSSDSSDSSDSSDSSDSSNSSNSSDSSNSSDSSDSSDSSDSSDSSDSSDSSDSSDSSDSSDSSDSSDSSNSSDSSNSSDSSNSSDSSNSSDSGDSSDSSNSSDSSDSSNSSDSSDSSNSSDSSDSSDSSDSSDSSDSSDSSDSSDSSDSSDSSDSSDSSDSSDSSDSSDSSDNDSSDSDSSDSSDSSDSSNSDSSNSSDSSESSDSSDSSDSSESSDSSDSSDSSESSDSSDSDSSDSSDSSDSSDSSDSSDSSNSSDSSDSDSSDSSDSSDSSNSSDSSDSDSSDSSDSSDSSNSSDSSDSSDSSDSSDSSNSSDSSNSSDSSDSSDSTSDSSDESDNQSKSGNGNNNGSDSDSDSEGSDSNHSTSDD*

**Gorilla**

MQGDDPNSSDESNGNDDANSESDNNSSSRGDASYNSDESKDNGNGSDSKGAEDDDSDSTSDTNNSDSNGNGNNGNDDNDKSDSGKGKSDSSDSDSSDSSNSSDSSDSSDSDSSDSNSSSDSSDSSDSDSSDSSDSDSSDSSNSSDSSDSSDSSDSSDSSDSKSDSDSSDSDSKSDSSDSNSSDSSDNSDSSDSSNSSNSSDSSDSSDSSDSSSSSDSSNSSDSSDSSDSSNSSDSSDSSDSSDSDSSDSSDSSNSSDSSNSNSSDSSDSSNSSDSSNSSDSSDSSNSSDSSDSSDSSNSSDSSDSSDSSDSSDSSDSSDSSDSSNSSDSNDSSDSSDSSDSSDSSNSSDSNHSSNSSDSSDSSNSSDSSDSSDSSDSDSSNSSDSSNSSDSSDSSNSSNSSDSSDSDSSNSSDNSNSSDSSNSSDSSDSSDSSDSNESSNSSDSSDSSNSSDSDSSDSSDSSDSSDSSDSSNSSDNSNSSDSSNSSDSSDSSDSSDSSNSSDSSNSSDSSNSSDSSDSNSSDSSDSSNSSDSSDSSDSSDSSDSSDSSDSSDSSDSSDSSDSSDSSDSSDSSDSSDSSDSSDRSNSSDSSNSSDSSNSSDSSDSSDSSDSSDSSDSSDSSDSSNSSDSSDSSDSSDSSDSSDSSDSSDSSDSSDSSNSSDSSDSSDSSDSSDSSDSDSSDSSDSSDSSDSSDSSDSSDSSDSSDSSDSSDSSDSSDSSDSSDSSDSSDSSDSSDSSDSSDSSDSSDSDSSDSSDSSDSSDSSDSSDSNESGDSSDSSDSSDSSDSSNSSDSSDSSDSSDSTSDSNDESDSQSKSGNGNNNGSDSDSDSEGSDSNHSTHDD*

**Human**

MQGDDPNSSDESNGNDDANSESDNNSSSRGDASYNSDESKDNGNGSDSKGAEDDDSDSTSDTNNSDSNGNGNNGNDDNDKSDSGKGKSDSSDSDSSDSSNSSDSSDSSDSDSSDSNSSSDSDSSDSDSSDSSDSDSSDSSNSSDSSDSSDSSDSSDSSDSSDSKSDSSKSESDSSDSDSKSDSSDSNSSDSSDNSDSSDSSNSSNSSDSSDSSDSSDSSSSSDSSNSSDSSDSSDSSNSSESSDSSDSSDSDSSDSSDSSNSNSSDSDSSNSSDSSDSSNSSDSSDSSDSSNSSDSSDSSDSSNSSDSSDSSDSSDSSDSSNSSDSNDSSNSSDSSDSSNSSDSSNSSDSSDSSDSSDSDSSNSSDSSNSSDSSDSSNSSDSSDSSDSSDGSDSDSSNRSDSSNSSDSSDSSDSSNSSDSSDSSDSNESSNSSDSSDSSNSSDSDSSDSSNSSDSSDSSNSSDSSESSNSSDNSNSSDSSNSSDSSDSSDSSNSSDSSNSSDSSNSSDSSDSNSSDSSDSSNSSDSSDSSDSSDSSDSSDSSNSSDSSDSSDSSDSSNSSDSSNSSDSSNSSDSSDSSDSSDSSDSSDSSDSSDSSNSSDSSDSSDSSDSSDSSDSSDSSDSSESSDSSDSSNSSDSSDSSDSSDSSDSSDSSDSSDSSDSSNSSDSSDSSDSSDSSDSSNSSDSSDSSESSDSSDSSDSSDSSDSSDSSDSSDSSDSSNSSDSSDSSDSSDSSDSSDSSDSSDSSDSSDSSDSSDSSDSSDSSDSSDSSDSNESSDSSDSSDSSDSSNSSDSSDSSDSSDSTSDSNDESDSQSKSGNGNNNGSDSDSDSEGSDSNHSTSDD*

**Chimpanzee**

MQGDDPNSSDESNGNDDANSESDNNSSSRGDASYNSDESKDNGNGSDSKGAEDDDSDSTSDTNNSDSNGNGNNGNDDNDKSDSGKGKSDSSDSDSSDSSNSSDSSDSSDSDSSDSNSSSDSDSSDSDSSDSSDSDSSDSSNSSDSSDSSDSSDSSDSSDSSDSKSDSSKSESNSSDSDSKSDSSDSNSSDSSDNSDSSDSSNSSNSSDSSDSSDSSDSSSSSDSSNSSDSSDSSDSSNSSESSDSSDSSDSDSRDSSDSSNSSDSSNSNSSDSSDSSNSSDSSDSSNSSDSSDSSDSSNSSDSSDSSDSSNSSDSSDSSDSSNSSDSSDSSDSSNSSDSSDSSDSSNSSDSSDSSDSSDSDSSNSSDSNDSSNSSDSSDSSNSSDSSNSSDSSDSSDSSDSDSSNSSDSSNSSDSSDSSNSSDSSDSSNSSDSSDSSDSSDSSDSDSSNSSDSSNSSDSSDSSDSSNSSDSSDSSDSSDSNESSNSSDSSDSSNSSDSDSSDSSDSSDSSDSNNSSDSNDSSNSDNSNSSDSSNSSDSSDSSDSSNSSDSSNSSDSSNSSDSSDSNSSDSSDSSNSSDSSDSSDSDSSDSSNSSDSSDSSDSSDSSDSSDSSNSDSSNSSDSSNSSDGSDSSDSSDSSDSSDSSDSSDSSDSSDSSDSSDSSDSSDSSDSSDSSDSSDSSDSSDSSDSSDSSDSSDSSESSDSSDSSDSSNSSDSSNSSDSSDSSDSSDSSDSSNSSDSSDGSDSSDSSDSSDSSDSSDSSDSSDSSDSSDSSDSSDSSDSSDSNESGDSSDSSDSSDSSDSSDSSDSSNSSDSSDSTSDSNDESDSQSKSGNGNNNGSDSDSDSEGSDSNHSTSDD*

**Armadillo**

MQGDDPNSSDESNDNNDANSKGDNNRSSREDNYKSDESKDNDNDSDSNGEEDDIDSNSTSDTNDSNDNGNNGSDNSKVDSSEGKLGSSDSSDSSDHSDSKSDSSENSESSDSDSKSDSSDSSDSKSDSSESSESSDSSDSDKSDSSDSKSDSSNSSDSSDSGSKSDSSDSKSDSSESSESSDSSDGSDGSDSDSKSDSRDSSDSSDSGSKSDSSESSDSSDSSDSKSDSSDSSETNSKSDSSDSDSKSDSNDSKPDSSDSSDSSDSNSKSDSSDRSESKSDSSDSSDSGNKSDSDSSDSSDSSESDSKSDNSDSSNSKSDSSDSSDSSESDSNDSSDSSESDSKSDSSDSSDSKSDSSDSSESDSKSDSSDSSDSSDSSDSKSDSSDSSDSSDSKSDSSDSSDNKSGSSDSSDSSDSSDNKSSSSGSSDSSDSKSGSSDSKSDSSDSSESDSKSDSSDSSDSSDSSDSSDSSDSSDSDSKSDSSVSDSDSSSNTSGNSDESDSKSESNGSYNRSDSDSDSNSEGSDSNSFDSDD*

**Tamandua Anteater**

MQGDDPNSRDESNGNDDANSESDDNSSSQGSSDGSDNSDNDNKSDSSDRANHSAVTVSWTAVTARQRAVTAVTVIANQTAGKQ*QQ***QQ*WNLQE*Q*QQITQQQQQ**L*EQ**QC****CQQ*QYQQQ*QQ*QQ***Q**WQL*Q*Q*QQITQSQQQ**Q*QQC*Q**SQQ*WKLQQ*L*QQITQ*QQQR*PWQQ*QQC*Q***QQ*WQL*Q*Q*QQITQQPQQQ*Q*QQQQ**QQ*QQLQQ*QQ***QQ*WQLQQ*Q*QQITQQQQQR*QWQQC*Q**QQQQQ*Q*QQ*QQRQQ*SQQQQDQK**QQQQ*Q*QQ*QCI*K***VAAGVSL*WQQQWK***H*Q*Q*RQRQ*SLNQ**LE

**Giant Anteater**

MQGDDPNS*DEPNGNDYANFESDNNSSSHGSSDSSDSSDNDNKSDRSDSESQSLSSDSKLDSSDSKSDSSDSSDSSDSDSSHDSYSSDSDSKSHSSNSGDSSDSSDSSDSVSSDSINSSYSSDSSDSDSKSHSHSSDSNDSSISSDSSNSSNNSDSNDSSDSSDSDGSNSSNSSDCSDSRDGSNSRTRSDSNNSSDSDSTDSASENSDEWQQEQVCNGSNNGSDSDFDSNSEAVTVITQPVMIR

**Manatee**

MQGDDPNSSDESNGNDDANSESDNNSRSRGDASYNSDESTDNDNDSDSKGDRDDAESDDSTPDANDSDSNGNGNNGSENNGKPESKSDSSDSGDSDSKSDSSESSDSSDSDSKSDSSESSDGDSSDSSDSSDSDSKSDSSESSDSSDSDSKSDSSESSDSDSSDSSDSSDSDSKSDSSESSDGDSSDTSDSSHSKSDSSDSGDNSDSKSDSSDSSDSSDSSDSDSKSDSSDSDSSDSSDSDSSDSSDSSESSDSDSKSDSSDSSDSSDSDSSDSSDSKSDSSDSSDSTASDSSDSKSESNDSDSKSDSSESSGSDSSDSSDSSHSKSDSSDSDSKSDSSDSSDSSDSKSDSSESSDSSDSDSNSDSSDSSGSDSSDSDSSDSSDSKSDSSDSSDSSESSDSDNKSDSSDSKSDSSDNSDSSDNSDSSDSSDSSDSSKSSDSSDSSDSSDSSDSNDSSNSSDSSDSSDSSNSSDSSDSSDGSDSSNSSDSSDSSDSSDSSDSSNSSVSSGSSSSSDSSDSSDSSDSDSSDSSNSSNSSDSSDSSDSSDNNSNDSTSDSSDESDSKSKSGYENNNGSDSDSDSDDSEGSDSNHSTSDD*

**Elephant African**

MQGDDPNSSNESNGNDDANSESENDSSSRGDDSYTSDESTDNGNDSDSKEEGDDAESDSTPDTNDGDSNGNGNNGNGNNGEPESKSDSSDSSDSSDSSDSSDSSDSSDSSDSSDSSDSKSDSSDSSDSSDSSDSSDSSDSSDSSDSSDSSDSSDSSDSSDSSDSSDSSDSSDSSDSSDSSNSSDSKSDSSDSSDSSDSSDSSDSSDSSDSSDSSDSSDSSDSSDSSKSSDSSDSSDSSDSSDSSDSSDSSDSSDSSDSSDSSDSSDSSDSSDSNDGSDSSDSSDSSDSSNSDSDSSDSSKSSDSSDSNDSSDSSDSTDSSDSSNSSDSSDSSDSNDSSDSSDNSGSNDSASNSSDESDSKSKSDNGNSNASDSDSDSEGSDSNHSTSDD*

**Elephant Asian**

MQGDDPNSSNESNGNDDANSESENDSSSRGDDSYTSDESTDNGNDSDSKEEGDDAESDSTPDTNDGDSNGNGNNGNGNNGEPESKSDSSDSSDSSDSSDSSDSSDSSDSSDSSDSSDSSNSSDSKSDSSDSSDSSDSSDSSDSSDSSDSSDSSDSSDSSKSSDSSDSSDSSDSSDSSDSSDSSDSSDSSDSSDSSDSSDSKSGSSDSSDSSDSSDSSDSSDSSDSSDSSDSSDSSDSSDSDSSDSNDGSDSSDSSDSSDSSNIDSSDSDSSDSSKSSDSSDSNDSSDSSDSTDSSDSSNSSDSSDSSDSNDSSDSSDNSGSNDSASDSSDESDSKSKSDNGNSNASDSDSDSEGSDSNHSTSDD*

**Opossum**

MQGDDPDSTDDDAQSESEDISNSKGNDSNEPDESIDDSNGNDSPEGGDEDSDSVSDTNDSDSTHNGSNSGDDSNGNGDSDSSDSKSDSDSKSDNNDSSDSSDSSDSSDSSDSSDSSDSSDSSDSSDSSDSSDSSDSSDSSDSSDSSDSDSKSDSSDSSDSSDSSDSSDSSDSDSKSDSSDSSDSSDSDSKSDSSDSSDSSDGDSKSDSSDSSDSSDSSDSSDSDSKSDSSDSSDSSDSDSKSESSDSSDNDSKSDSSDSSDSSDSSDSSDSDSKSDSSDSSDSSDSSDSSDSSNSDSKSESSDSSDSSDSDSKSDSSDSSDSDSKSDSDSKSESSDSSDSDSESDSSDSSDSSDSDSKSDSDSKSDSSDSSDSSDSSDSDSKSDSSDSSDSDSKSDSSDSSDSSDSDSKSDSSDSSDSDSKSDSSDSSDSDSESDSSDSSDSSDSDSKSDGDNNNHQGKYNNRNDSDSDSDSDSSVSEGSDSNHSTSDD*

**Platypus**

MQGDDPDSSDETNGSDSSQQAGNEGAESESENASNSEGHLSSNSDESNGGGDDDGDGDDDSDSKSDGGDSNGKSDVNGGSDDSDSKSDSSNSSDSSDSNDSSESKSDSSNSSDSSDSSDSSDSCDSSDSSDSKSDSSNSSNSSDSSDSSDSSDSKSDSSDSSDSSDSSNSKSDSSDSSDSSSNSSDSKSDSSDSSDSSDSSDSKSNSSNSSDSDSKSDSSDSDSSDSSDSSDSKSDSSDSSDSSDSSDSKSDSSDSSDSSDSSHSKSDSSDSSDSNKSKSDSSDSCESSESKSDSNDSSDSSDSSESKSDSSDSSDSSDSSDSKSDSSDSSDSSDSSDSKSDSSDSSDSSNNSDSKSDSSDSSDSSDSSDSKSDSDSKSDSSDSSDSRNSKSDSSDSSDSSDNNDSKSDGNDSSDSSDSKSDSSNSSDNTSDSSDSSESKSNSSDNTSENTNESDGDKSSSSTSNGSDSELEEQNDNSNAKVVDDDSDSASDSNNSTSDEGRIRLNLQD*

**Anole**

MQGDDPGYSDNSDSSQQARGQSDSGHQPGNSDSPNSLNEEETEQFTTSVESGVLNASSRSPDSSA**S**SD**S**GT**S**SD**S**CN**S**RN**S**NDCSG**S**SHASS**S**SS**S**SD**S**NDSND**SS**D**S**SD**S**SN**SS**D**S**SD**S**SDT**S**E**S**NS**S**SD**S**SDLRD**SS**E**S**GS**S**SE**S**SD**S**NE**S**KD**S**SE**S**RD**SS**D**S**SD**S**SDT**S**E**S**NS**S**SD**S**SD**S**STF**S**KTSS**S**SE**S**RD**S**SD**S**RD**SS**D**S**SD**S**SHT**S**E**S**SS**S**SE**S**AD**S**SESRD**SS**D**S**SE**S**SDA**S**E**S**KS**S**SD**S**SD**S**RE**S**PE**S**SS**S**SE**S**RD**S**SN**S**AD**SS**D**S**NS**S**SD**S**SI**S**SN**SS**I**S**SD**S**SD**S**NS**SS**Y**S**RN**S**ND**S**SD**S**LTN**S**SHQSD**S**NS**S**NA**S**ST**S**NNSNDTSSDSGRPSSSQINCFENYHKIPHICEEYISVQTTVT*
